# Strength of shear bands in fluid-saturated rocks: a nonlinear effect of competition between dilation and fluid flow

**DOI:** 10.1038/s41598-018-19843-8

**Published:** 2018-01-23

**Authors:** Evgeny V. Shilko, Andrey V. Dimaki, Sergey G. Psakhie

**Affiliations:** 1 Institute of Strength Physics and Materials Science SB RAS, Laboratory of Computer-Aided Design of Materials, Tomsk, 634055 Russia; 2 Tomsk State University, Faculty of Physics, Tomsk, 634050 Russia; 3 Tomsk Polytechnic University, Institute of High Technology Physics, Tomsk, 634050 Russia

## Abstract

This study shows the significant and nonlinear effect of the competition between dilation and fluid flow on the shear strength of constrained shear bands in fluid-saturated rocks. This effect is conditioned by the contribution of the pore pressure to the yield stress and strength. The pore pressure is controlled by the dilation of the pore space in the solid skeleton of the shear band during plastic deformation and by squeezing of pores in surrounding blocks by the dilating shear band due to the high stiffness of the host massif. A generalized equation has been derived to describe the dependence of the shear band strength on the ratio of strain rate to fluid flow rate.

## Introduction

A range of laboratory and full-scale geological and geophysical research suggests that irreversible deformation in rock samples and rock massifs is strongly localized in shear bands at different scales, the largest of which are tectonic faults^1–3^. These narrow zones not only determine the compliance of rocks in the form of localized relative shear displacements of structural blocks, but control seismic activity of rock massifs. The latter explains the current interest in the mechanical properties of fault zones and the rapid increase in the number of published works in this area.

One of the key mechanical properties is the maximum (or peak) strength of the fault zone under given stress and confinement conditions^4–7^. Reaching the maximum strength corresponds to a change in the response of the shear band from pervasive strain (strain hardening stage) to strain localization (strain softening stage)^8,9^. The behavior of the shear band at the strain softening stage is largely determined by the degree of consolidation of the gouge. In particular, for shear bands with a mature zone of unconsolidated/granular gouge the hardening-to-softening transition is typically smooth and does not lead to onset of dynamic slip as granular gouge usually exhibits velocity-strengthening frictional behavior^10–12^. At the same time, consolidated (lithified and indurated) shear bands behave in a fundamentally different way with rapid stress drop (corresponding to brittle rupture) and transition to dynamic slip accompanied by radiation of elastic waves (velocity-weakening behavior)^9,10,13^. The radiated acoustic/seismic power is determined by the magnitude of the dynamic stress drop, which in turn strongly depends on the peak strength of the shear band. This problem is especially important for faults or fault segments with healed (consolidated) core zones, which typically have high shear strength. Failure of such faults or fault segments has a dynamic character and is accompanied by generation of strong seismic waves^14–16^. Therefore, the estimation of maximum shear strength is both a fundamental and practical problem widely discussed in fault and rock mechanics^4–7^.

The conditions for onset of pervasive inelastic strain and subsequent reaching of the maximum strength of shear bands (including faults) are mainly affected by the pore structure and pore fluid pressure. The pore pressure dynamics is controlled by two interrelated processes^17–27^: (1) fluid flow and (2) pore volume change. The pervasive inelastic deformation of rock is often accompanied by its dilatancy^28–30^. The volume of the connected crack-pore space in the rock increases during pervasive shear deformation of the shear band, which leads to decrease of local pore pressure. The reduction of fluid pressure reduces the intensity of the relaxation processes associated with the formation of new discontinuities and coalescence of existing ones. This effect is called dilatancy hardening^18^. In turn, fluid inflow is able to compensate for the pore pressure drop and reduce the effect of strain hardening^27,31^. The ratio of fluid flow rate to strain rate (which governs the dilation rate) determines the specific value of shear strength of shear bands. Note that the influence of the competition between dilatancy and fluid flow on shear strength is strongly pronounced for shear bands that are surrounded by material blocks with a similar permeability to that of the shear band gouge. This particularly corresponds to healed (consolidated) faults where the difference in the porosity and permeability of the principal slip zone (of width 1–10 cm) and surrounding periphery zone (up to several meters wide) is much less pronounced than in faults with a mature zone of unconsolidated gouge^32^.

Conventionally, the effect of pore fluid on the maximum shear strength (hereinafter referred to as strength) of shear bands, including fault zones, has been studied for limiting modes of deformation (very slow and very fast) that correspond to drained and undrained hydrological conditions^33^. This is principally due to the fact that the limiting modes most closely correspond to the long-term creep and short-term dynamic modes of deformation of fault zone regions. Numerous experimental and theoretical works, starting with the classical paper of Brace and Martin^33^, show that the strength of permeable rock samples can increase significantly (up to 30–50%) in transition from the drained to undrained condition^31,34–40^. This is explained by the limitation of fluid inflow to the increasing pore space of the incipient shear band and corresponding inhibition of pore pressure drop recovery.

Despite the progress achieved in studying the mechanical behaviour of shear bands (including fault zones) in permeable rocks, the influence of some essential factors is not fully understood. Among them are permeability of the shear band and the blocks and their “fluid holding capacity” (which depends on porosity and size). Another important factor is the elastic stiffness of the bulk of rock surrounding the shear band^12,41^. This factor is traditionally associated with the magnitude of effective normal stress defined as the difference between applied normal stress and local pore pressure^9,27,42^. The magnitude of elastic stiffness reaches its maximum value in the bulk of the rock massif, where the mechanical constraint by surrounding rock strongly limits movement of blocks adjacent to the shear band in the normal direction. In such conditions, dilation of the shear band or core zone of the fault causes progressive compression of adjacent block regions and is accompanied by increase in the value of compressive volumetric stress (and compressive effective normal stress) in the fault zone.

There is still no unambiguous understanding of how the strength of a shear band in the depth of constrained permeable rock massif changes in the transition region between the undrained and drained conditions. The topicality of this question is particularly related to the important and widely discussed problem of using watering to control the dynamics of shear stress relaxation in fault zones^27,43,44^. Keeping this question in mind, we studied the nature and functional form of the dependence of the shear band strength on the ratio of shear strain rate to fluid flow rate under constrained conditions corresponding to faults in rock massifs. The study was performed by numerical modelling of the shear deformation of a fluid-saturated permeable shear band using the discrete element method.

## Problem Statement

**Figure 1:**
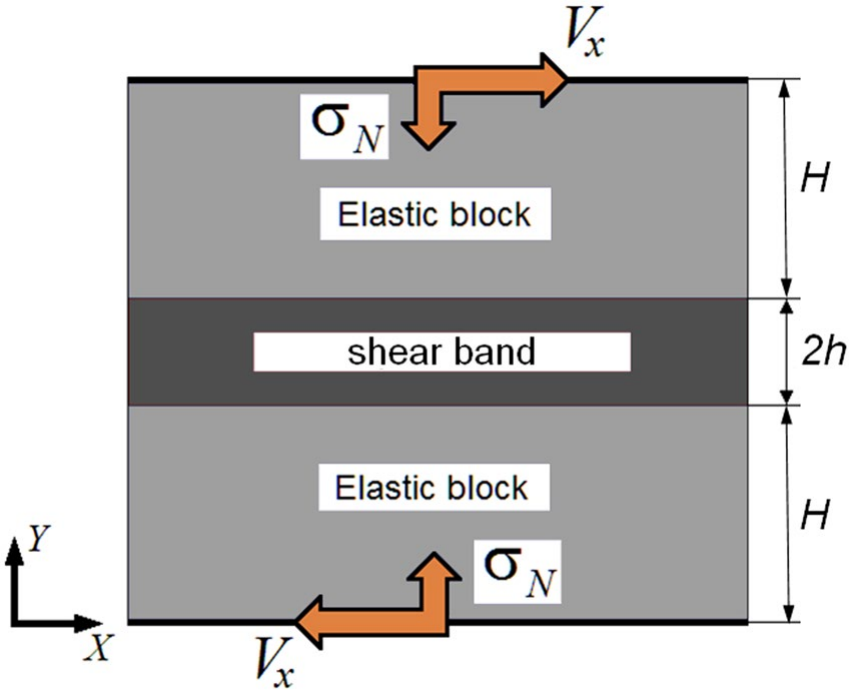
Model sample structure and loading scheme for modelling of constrained shear of a porous fluid-saturated shear band surrounded by porous fluid-saturated blocks.

We considered a 2D model sample consisting of two blocks separated by an interfacial layer (shear band) in the plane strain approximation (Fig. 1). The blocks imitate the regions of the medium adjacent to the shear band, which are less damaged than the shear band, have higher cohesion, and therefore deform elastically under the considered loading conditions. The shear band of width 2 *h* is a layer of an elastic-plastic dilatant material, which simulates the layer of consolidated gouge in principal slip zones of faults^9,45^. The width of the model blocks was *H = *20 *h*. We used the following reference values of widths of the shear band and blocks: 2 *h* ≈ 1.5 cm, *H* = 15 cm. The shear band and blocks were assumed to be permeable and fluid saturated.

The model shear band with surrounding fragments of blocks was numerically simulated by the discrete element method^46–49^ using a fully coupled macroscopic model of fluid-saturated porous brittle materials^50,51^. Within this model the discrete elements simulating parts of the shear band and surrounding blocks are treated as porous and permeable. The effect of the fluid contained in the crack-pore space of a discrete element on its stress state is described on the basis of the Biot linear model of poroelasticity^52–54^. The inelastic behavior of the permeable brittle material of the discrete element is described using a plastic flow model of rocks with a non-associated flow law and the Mises–Schleicher yield criterion (Nikolaevsky’s model)^55,56^:

1 $${\rm{\beta }}{\sigma }_{mean}^{eff}+\frac{{{\rm{\sigma }}}_{eq}}{\sqrt{3}}={\rm{\beta }}({{\rm{\sigma }}}_{mean}+{P}_{pore})+\frac{{{\rm{\sigma }}}_{eq}}{\sqrt{3}}=Y,$$

where β and *Y* are the internal friction coefficient and cohesion (yield stress under pure shear deformation) of dry material of the discrete element, *P*_*pore*_ is the pore pressure, σ_*mean*_ and σ_*eq*_ are the mean stress and the equivalent stress within the discrete element volume^51^. Nikolaevsky’s model assumes a linear relation between the rates of shear and bulk plastic strains with the dilatancy factor Λ. The dilation of the material of the discrete element leads to an increase in its porosity and permeability^51^. Within the formalism of discrete elements, local failure is modeled by changing the state of a pair of interacting elements from linked (or bonded) to unlinked^49^. As the failure criterion we apply the Drucker–Prager criterion in the following formulation:

2 $$1.5(\lambda -1)({{\rm{\sigma }}}_{mean}+{P}_{pore})+0.5(\lambda +1){{\rm{\sigma }}}_{eq}={{\rm{\sigma }}}_{c},$$

where λ = σ_*c*_/σ_*t*_ is the strength ratio of “dry” material under uniaxial compression (σ_*c*_) and tension (σ_*t*_). The pressure-dependent Mises-Schleicher and Drucker-Prager criteria are generalizations (smoothing) of the Mohr-Coulomb yield and failure criteria, which are widely used in rock and fault mechanics^4,7,24,38,57^. The influence of the pore fluid pressure *P*_*pore*_ on the conditions of onset of inelasticity and fracture is taken into account within these criteria by means of their formulation in terms of effective mean stress ${{\rm{\sigma }}}_{mean}^{eff}={{\rm{\sigma }}}_{mean}+{P}_{pore}$.

The elastic characteristics of the shear band and blocks were assumed to be similar and corresponded to typical values for sandstones with a porosity of 10–15% (Young’s modulus *E* = 15 GPa, Poisson’s ratio ν = 0.3). The material of discrete elements modelling the blocks was treated as elastic-brittle and high-strength. The material of discrete elements modelling the shear band was a model elastic-plastic material with linear hardening with the following plasticity and strength parameters: β = 0.57, Λ = 0.36, *Y* = 10.84 MPa (this corresponds to a yield stress of 28 MPa under uniaxial compression), strain hardening modulus Π = 515 MPa, uniaxial compressive strength σ_*c*_ = 40 MPa, uniaxial tensile strength σ_*t*_ = 13.33 MPa (λ = 3). The calculations were carried out at an initial mean stresses ${{\rm{\sigma }}}_{mean}^{0}$ below the brittle-ductile transition threshold (in this case, at ${{\rm{\sigma }}}_{mean}^{0} < 40\,{\rm{MPa}}$).

The initial values of porosity (ϕ_0_ = 0.1) and permeability *k*_0_ of the shear band and the blocks were assumed to be equal. This approximation is consistent with the low gradients of porosity and permeability in central zones of healed (consolidated) faults.

The model sample was simulated by a close-packed ensemble of equally sized discrete elements. Initially, all interacting elements were assumed to be linked to imitate a consolidated shear band. The element size was 5·10^−4^ m. Separate tests have shown that further reduction of the element size does not have any significant effect on the simulation results (see Supplementary Materials).

We modelled constrained shear of the sample in the horizontal plane along the *X* axis (Fig. 1). Periodic boundary conditions were specified on the lateral faces in the horizontal direction to simulate an infinitely long shear band. The sample was loaded in two stages. At the first stage, a normal load σ_*N*_ was applied to the upper and lower sample faces. The initial fluid concentration in the pore space of the sample was chosen so as to create the specified pore pressure ${P}_{pore}^{0}$ in the sample. There was no plastic deformation in the sample by the end of the first loading stage. The stress and pore pressure distributions were homogeneous. At the second stage, the sample was subject to simple shear by applying constant tangential velocity *V*_*x*_ and zero normal velocity (along the *Y* axis) to the upper and lower faces to fulfil the constrained shear condition. The sample deformation proceeded until crack initiation in the shear band.

The described 2D system models a horizontal cross section of a healed fault between structural blocks of a rock massif at a certain depth. Note that the initial “horizontal” (in the *XY* plane) stresses in the given formulation of the problem exceed the “vertical” ones. This is consistent with experimental data indicating that horizontal stresses are considerably higher than vertical ones in regions with high deformation activity^58^.

We used the following types of hydrological boundary conditions on the external surfaces of the sample: (1) impermeable boundaries (hydraulically isolated sample) or (2) perfectly permeable (pore pressure on the surfaces was permanently equal to the initial value ${P}_{pore}^{0}$). These boundary conditions correspond to the hydrological conditions in the central regions of fault zones in the bulk of low and high permeability host rocks, respectively.

## Results

**Figure 2:**
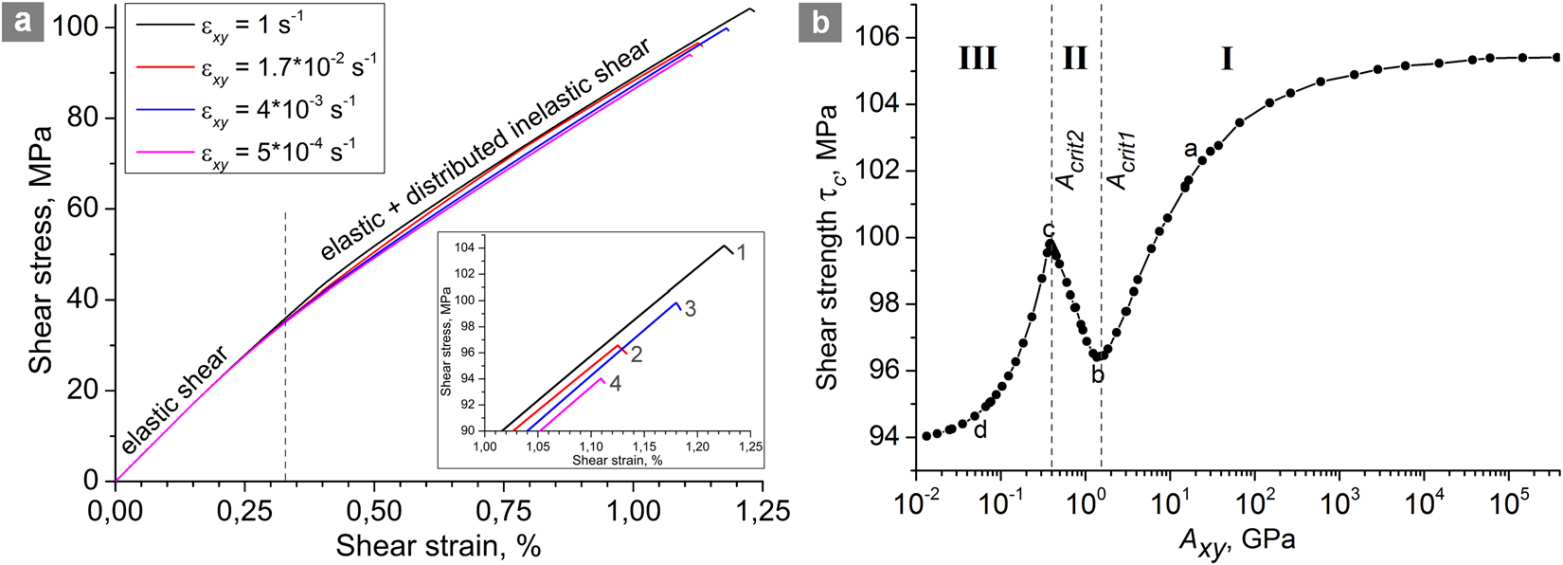
Shear strength variability. (**a**) Shear loading curves of the reference sample (2 *h* ≈ 1.5 cm, *H* = 15 cm) at different shear strain rates ${\dot{{\rm{\varepsilon }}}}_{xy}$. The values of permeability (*k*_0_ = 10^−15^ m^2^) and fluid viscosity (η = 10^−3^ Pa·s) are the same for all curves. (**b**) A typical dependence of the shear strength τ_*c*_ of the shear band on the parameter *A*_*xy*_ for a hydraulically isolated sample (*h*/*H* = 0.05, ${P}_{pore}^{0}=0.5|{{\rm{\sigma }}}_{mean}^{0}|=17.5\,{\rm{MPa}}$). Roman numerals I–III mark the curve regions corresponding to different behavior modes of the fluid-saturated sample in shear. The values *A*_*crit1*_ and *A*_*crit2*_ correspond to the local minimum and maximum shear strength. Points a–d indicate the values of *A*_*xy*_ for which the pore pressure and mean stress distributions are shown in Fig. 3. The top and the bottom faces of the sample are fixed in vertical direction. Lower *A*_*xy*_ imply faster fluid flow or lower strain rate, higher *A*_*xy*_ imply slower fluid flow or higher strain rate.

Figure 2a shows examples of shear loading curves of the reference sample (2 *h* ≈ 1.5 cm, *H* = 15 cm) at different shear strain rates. Here shear stress is calculated as the ratio of shear resistance force, measured at the upper sample face, to the square of the face. It can be seen that the initially elastic deformation transitions into inelastic deformation distributed in the volume of shear band (pervasive shear strain accompanied by dilation). Upon reaching the maximum shear stress (which is a peak shear strength) the shear crack is nucleated and developed in the shear band along the direction of band strike.

The simulation results showed that at high strain rates the magnitude of shear strength tends toward the upper limit, and at low strain rates – toward the minimal value. In the example shown in Fig. 2a the values of shear strength for curves 1 and 4 are close to the upper and lower limits, respectively. Such regularity was first observed by Brace and Martin^33^, and then reported in numerous experimental and theoretical studies of confined compression of rock samples and shear loading of model fault zones^31,34–39^. At the same time, we found that, in general, the reduction of shear strength from the upper to the lower limit with reduction of strain rate is not monotonous. At a certain intermediate strain rate the shear strength reaches a local minimum (curve 2 in Fig. 2a). Further reduction of strain rate leads to an *increase* of shear strength up to a local maximum (curve 3 in Fig. 2a). At even smaller strain rates the shear strength again *decreases* down to the lower limit. This result was unexpected, but is confirmed by recent experimental studies^31,40,59–62^.

In the present study, we varied the shear strain rate ${\dot{{\rm{\varepsilon }}}}_{xy}={V}_{x}/(h+h)$, the initial permeability of the blocks and the shear band *k*_0_ (at fixed ϕ_0_ = 0.1), the dynamic fluid viscosity η, and system size (2*H* + 2*h*) within several orders of magnitude: ${\dot{{\rm{\varepsilon }}}}_{xy}$ from 5·10^−4^ s^−1^ to 1 s^−1^, *k*_0_ from 10^−18^ m^2^ to 10^−13^ m^2^, η from 2·10^−4^ Pa·s to 2·10^−2^ Pa·s (dynamic viscosity of water at room temperature is about 10^−3^ Pa·s), (2*H* + 2*h*) from 15 cm to 150 cm. We found that the parameter combination

3 $${A}_{xy}={\dot{{\rm{\varepsilon }}}}_{xy}{\rm{\eta }}{(H+h)}^{2}/{k}_{0},$$

unambiguously determines the value of shear strength of the shear band for a given initial mean stress ${{\rm{\sigma }}}_{mean}^{0}$, pore pressure ${P}_{pore}^{0}$, and ratio *h*/*H*. In other words, shear band zones have the same shear strength if they are characterized by the same value of *A*_*xy*_, (even if the specific values of the parameters *k*_0_, η, ${\dot{{\rm{\varepsilon }}}}_{xy}$, and *h* differ by orders of magnitude). The parameter *A*_*xy*_ has the meaning of strain rate normalized by fluid flow rate, because the combination *k*_0_/η(*H* + *h*)^2^ detеrmines the fluid flow rate in conventional equations of fluid mass transfer.

Figure 2b shows a typical dependence of the shear strength τ_*c*_ of the modelled shear band on the parameter *A*_*xy*_ for a hydraulically isolated system. Each point of the curve corresponds to a separate calculation at given values of *k*_0_, η, ${\dot{{\rm{\varepsilon }}}}_{xy}$ and *h* (at *h*/*H* = *const*, σ_*N*_ = *const*, ${P}_{pore}^{0}=const$). The region *A*_*xy*_ → ∞ (region I in Fig. 2b) corresponds to combinations of *k*, η, ${\dot{{\rm{\varepsilon }}}}_{xy}$ and *h* at which the fluid flow rate is extremely low compared to the rate of pore pressure change caused by pore volume variation. This corresponds to the hydrological conditions close to the undrained condition of the shear band. The region of low *A*_*xy*_ values (region III in Fig. 2b) corresponds to low shear rate, low fluid viscosity or high permeability of the blocks. In this region the fluid flow rate is relatively high, and the hydrological conditions for the shear band approach a fully drained (the pore pressure distribution in the sample is close to homogeneous during the entire course of deformation).

**Figure 3:**
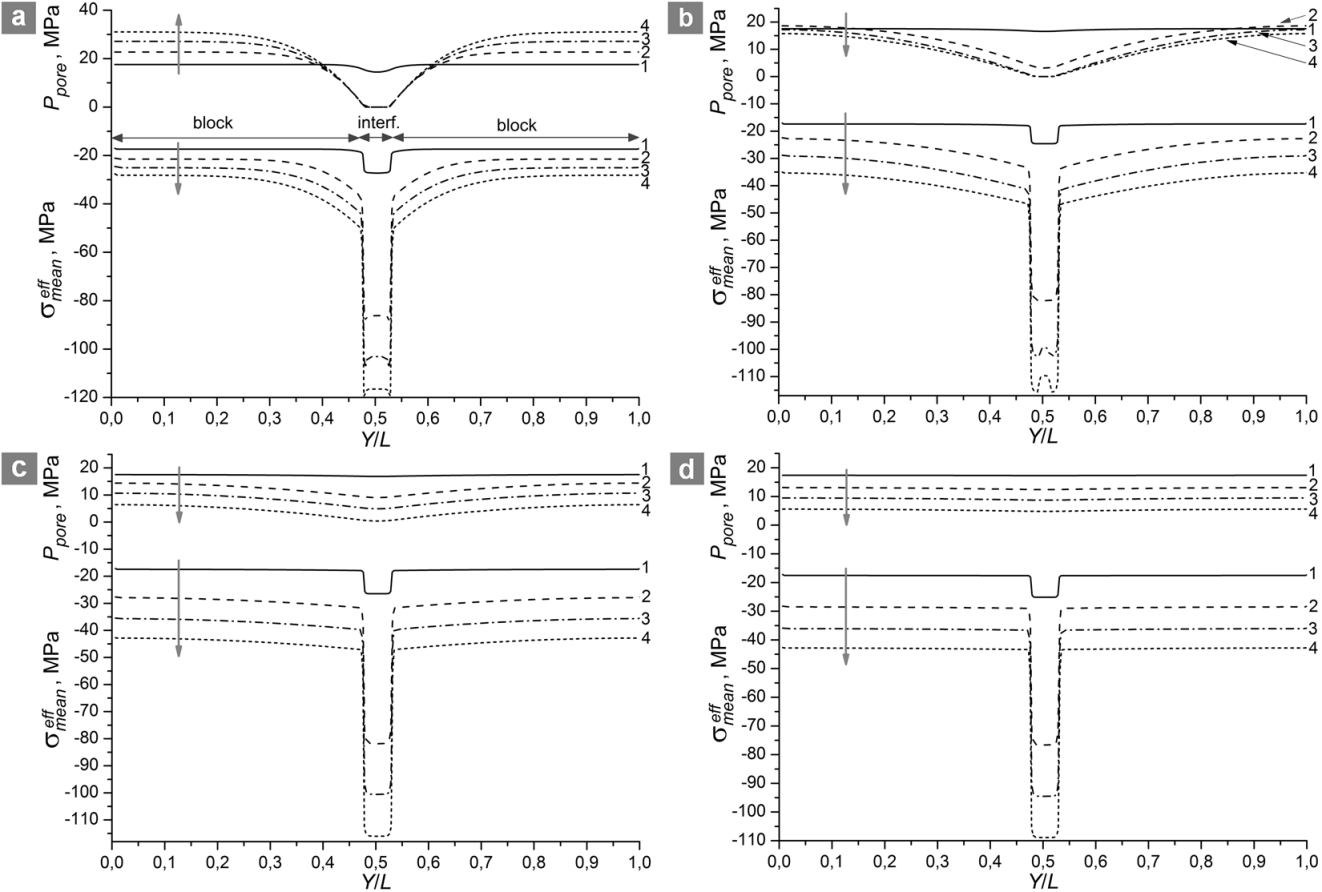
Distributions of pore fluid pressure *P*_*pore*_ and effective mean stress ${{\rm{\sigma }}}_{mean}^{eff}$ in the *Y* direction in fluid-saturated samples (*h*/*H* = 0.05, ${P}_{pore}^{0}=0.5|{{\rm{\sigma }}}_{mean}^{0}|=17.5\,{\rm{MPa}}$) for different *A*_*xy*_ values: (**a**) 24 GPa; (**b**) 1.5 GPa; (**c**) 0.384 GPa; (**d**) 0.0496 GPa. Four pairs of curves in each figure correspond to different values of applied shear strain ε_*xy*_: 0.2 ${{\rm{\varepsilon }}}_{c}^{{A}_{xy}}$ (1); 0.6 ${{\rm{\varepsilon }}}_{c}^{{A}_{xy}}$ (2); 0.8 ${{\rm{\varepsilon }}}_{c}^{{A}_{xy}}$ (3); 0.98 ${{\rm{\varepsilon }}}_{c}^{{A}_{xy}}$ (4). Here ${{\rm{\varepsilon }}}_{c}^{{A}_{xy}}$ is the ultimate (at the moment of failure) value of applied shear strain for the corresponding sample. The arrows indicate the direction of the flow of time (direction of shear strain increase). The samples are hydraulically isolated; their top and bottom faces are fixed in vertical direction. Lower *A*_*xy*_ imply faster fluid flow or lower strain rate, higher *A*_*xy*_ imply slower fluid flow or higher strain rate.

The strength τ_*c*_ of the shear band approaches the absolute maximum at *A*_*xy*_ → ∞ (dynamic loading, highly viscous fluid or impermeable blocks) and tends to the absolute minimum at *A*_*xy*_ → 0 where permeability of the sample is large enough to provide homogeneous distribution of pore pressure at all stages of deformation. At the same time, the dependence τ_*c*_(*A*_*xy*_) in the interval of intermediate *A*_*xy*_ values (region II in Fig. 2b) has unexpected non-monotonic character. The non-monotonicity of the τ_*c*_(*A*_*xy*_) dependence can be explained by analysing the dynamics of pore pressure and effective mean stress distributions across the vertical section of the samples (Fig. 3).

At high *A*_*xy*_, the pore fluid pressure in the shear band drops to zero at the early stage of inelastic deformation accompanied by dilation (curves 1–4 for *P*_*pore*_ in Fig. 3a). Due to the mechanical confinement of the system, shear band expansion leads to progressive compression of the blocks, which in turn causes increased compressive mean stress and pore pressure (at the same time, the magnitude of expansion is smaller than in an unconstrained system). Consequently, the absolute value of effective mean stress within the shear band increases during the course of deformation (effective mean stress is compressive, i.e. negative). The magnitude of the effective mean stress in the shear band is up to several times higher than in bordering regions of blocks, and this difference gradually increases with shear strain (curves 1–4 for ${{\rm{\sigma }}}_{mean}^{eff}$ in Fig. 3a). The maximum absolute value of effective mean stress in the shear band is achieved for the case of negligible fluid flow rate, which results in the maximum shear strength at *A*_*xy*_ → ∞ (Fig. 2b).

**Figure 4:**
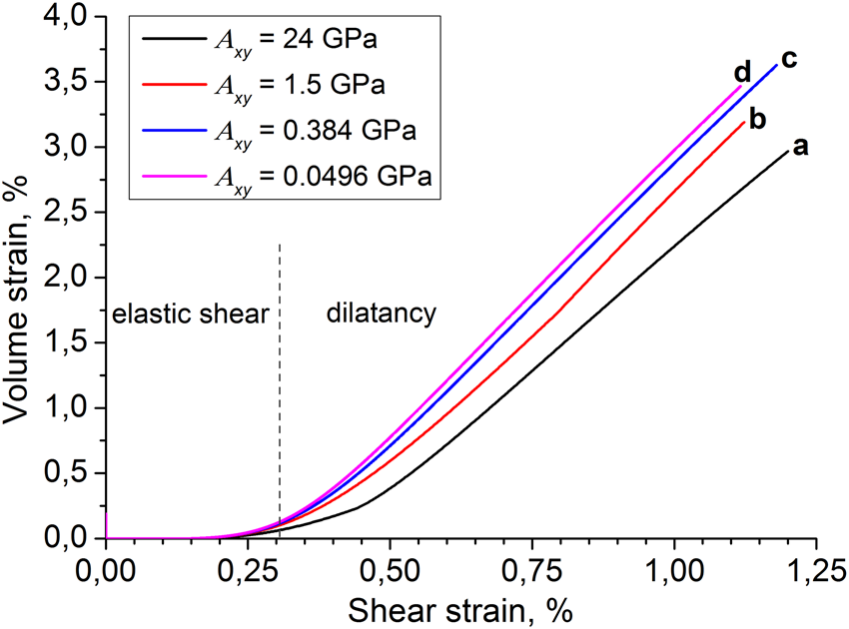
The dependences of the shear band volume strain on applied shear strain for a hydraulically isolated sample (*h*/*H* = 0.05, ${P}_{pore}^{0}=0.5|{{\rm{\sigma }}}_{mean}^{0}|=17.5\,{\rm{MPa}}$) characterized by different *A*_*xy*_ values: 24 GPa (**a**); 1.5 GPa (**b**); 0.384 GPa (**c**); 0.0496 GPa (**d**). The volume strain was determined as the change of the shear band width Δ*h* from its initial value 2 *h*. Curves a–d correspond to the same values of *A*_*xy*_, as in Figs 2d and 3. The top and the bottom faces of the samples are fixed in vertical direction. Lower *A*_*xy*_ imply faster fluid flow or lower strain rate, higher *A*_*xy*_ imply slower fluid flow or higher strain rate.

A reduction of the parameter *A*_*xy*_, for example as a result of a decrease in strain rate or viscosity or an increase in the permeability, leads to increase in the rate of fluid flow from the blocks to the shear band. Moreover, at sufficiently low values of *A*_*xy*_, the pore pressure in the blocks decreases (instead of increasing) under shear deformation (curves 1–4 for *P*_*pore*_ in Fig. 3b), despite the dilation of the interface and block compression. Fluid outflow is accompanied by a decrease in the linear dimensions of the blocks. The latter determines the increase in the rate of shear band expansion (curves a and b in Fig. 4) and the corresponding decrease in the absolute values of effective mean stress in the shear band and blocks as compared to an impermeable system (curves 1–4 for ${{\rm{\sigma }}}_{mean}^{eff}$ in Fig. 3b). Besides this, due to increase of fluid inflow, a substantial gradient of effective mean stress forms in the cross section of the shear band. The minimum of ${{\rm{\sigma }}}_{mean}^{eff}$ is localized at the center. The described changes in the stress state cause a decrease in the strength τ_*c*_ of the shear band with decreasing *A*_*xy*_ (region I in Fig. 2b). At a certain “threshold” combination of the filtration and deformation parameters *A*_*xy*_ = *A*_*crit*1_, the shear strength τ_*c*_ reaches its local minimum (point “c” in Fig. 2b).

In the region *A*_*xy*_ < *A*_*crit*1_ (region II in Fig. 2), the ratio of fluid flow rate to strain rate becomes large enough to maintain a nonzero fluid pressure in the shear band up to large values of applied shear strain ε_*xy*_ (curves 1–4 for *P*_*pore*_ in Fig. 3c), including the point of failure. In this case, the smaller is the value of *A*_*xy*_, the higher is the pore pressure in the shear band at the same value of applied shear strain ε_*xy*_. As the pore pressure rises, the inelastic contribution to the total strain rate increases. Consequently, the dilation rate increases with decrease of *A*_*xy*_. This effect is most pronounced at the early stage of inelastic strain (curves b and c in Fig. 4). In the interval of *A*_*xy*_ corresponding to region II in Fig. 2b the increase in the rate of block compression by the dilating shear band overcompensates for the contraction of blocks caused by fluid outflow. As a result, in region II the effective mean stress in the shear band again increases with decrease of *A*_*xy*_ (curves 1–4 for ${\sigma }_{mean}^{eff}$ in Fig. 3c). Moreover, the gradient of effective mean stress in the cross section of the shear band decreases at the same time. The above considerations explain the observed increase in the strength τ_*c*_ of the shear band. At some “threshold” combination of the filtration and deformation parameters *A*_*xy*_ = *A*_*crit*2_, the shear strength τ_*c*_ reaches its local maximum (point “c” in Fig. 2b).

In the region *A*_*xy*_ < *A*_*crit*2_ (region III in Fig. 2), which corresponds to an extremely low rate of applied shear strain or high permeability, the fluid flow rate provides nearly homogeneous distributions of pore pressure across the sample and of effective mean stress across the blocks and the shear band (Fig. 3d). A key feature of region III is that the pore pressure in the shear band remains nonzero up to the moment of crack formation and its value increases with decrease in *A*_*xy*_ (*P*_*pore*_ ≈ 5 MPa or about 30% of the ${P}_{pore}^{0}$ value in the example shown in Fig. 3d). The threshold *A*_*crit2*_ corresponds to a combination of the parameters at which the pore pressure in the center of the shear band reaches zero at the moment of crack initiation. The increase of dilation rate with increasing *P*_*pore*_ tends to saturation in region III (curves c and d in Fig. 4). Therefore, increase of the pore pressure in the shear band in region III leads to slower growth of effective mean stress during the course of deformation and hence to decrease in the shear strength. The lower limit τ_*c*_ is reached at *A*_*xy*_ → 0 (fully drained condition for the shear band).

**Figure 5:**
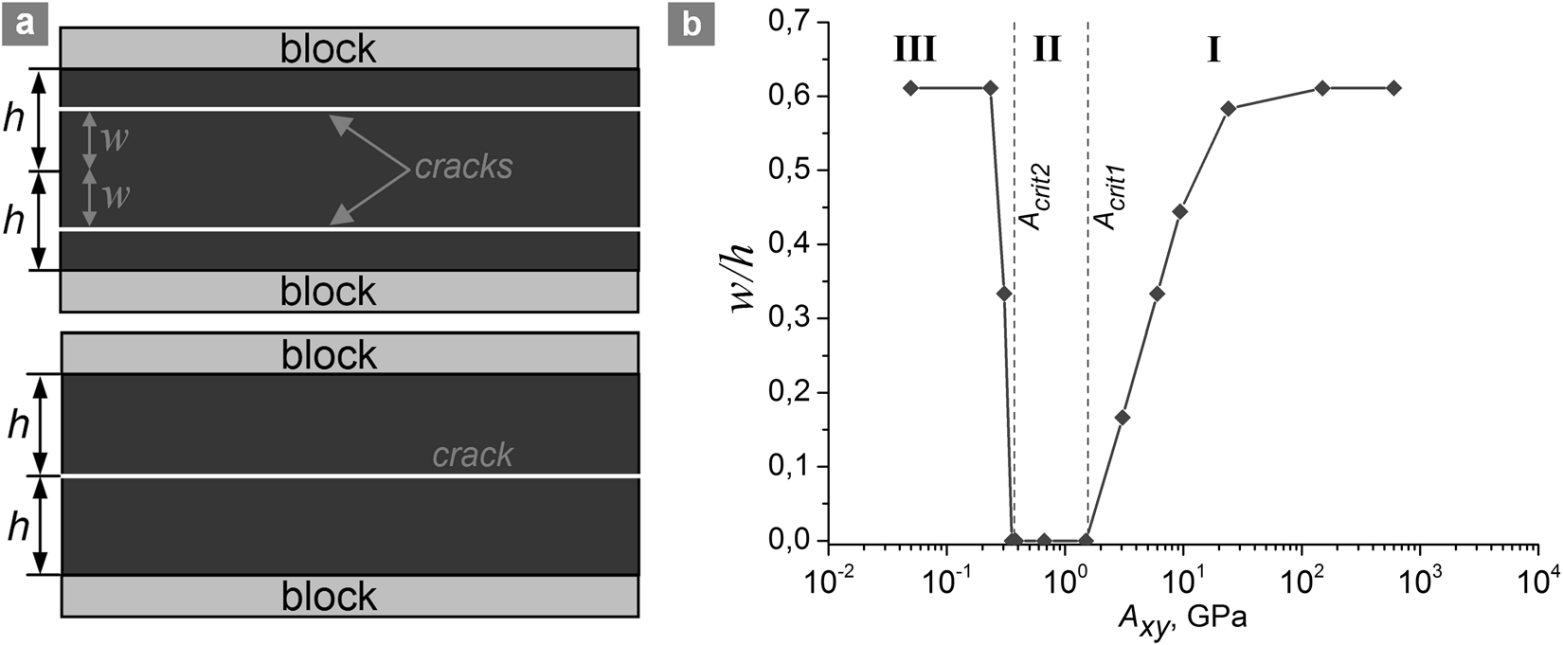
Crack line position variability. (**a**) Schemes of crack location in the periphery (upper picture) and in the centre (lower picture) of the shear band. (**b**) The dependence of normalized distance from crack to the centre of shear band on the parameter *A*_*xy*_ for a hydraulically isolated sample (*h*/*H* = 0.05, $${P}_{pore}^{0}=0.5|{\sigma }_{mean}^{0}|$$$$=\,17.5\,{\rm{MPa}}$$). Roman numerals I–III mark the curve regions corresponding to different behavior modes of the fluid-saturated sample under shear loading. The values *A*_*crit1*_ and *A*_*crit2*_ correspond to the local minimum and maximum shear strength (Fig. 2b). The top and the bottom faces of the sample are fixed in vertical direction. Lower *A*_*xy*_ imply faster fluid flow or lower strain rate, higher *A*_*xy*_ imply slower fluid flow or higher strain rate.

The described qualitative change of the distribution of effective means stress also determines the position of the shear crack in the shear band (Fig. 5). At large and small values of *A*_*xy*_ (*A*_*xy*_ ≫ *A*_*crit*1_ and *A*_*xy*_ < *A*_*crit*2_, respectively) a pair of symmetrically positioned cracks is formed (formation of a pair of cracks is a consequence of the symmetry of the system). The distance between the cracks and the center of the shear band is approximately 60% of the half-width of the shear band (the upper scheme in the Fig. 5a). In the region I the crack position gradually shifts to the center of the shear band following the decrease in strength of the shear band with decrease of *A*_*xy*_ (Figs 2b and 5b). The interval of *A*_*xy*_, where crack shifts from “limiting” position (60% of the half-width of the shear band) to the center of the shear band, approximately amounts to two orders of magnitude. In the region II (within the range *A*_*crit*2_ < *A*_*xy*_ < *A*_*crit*1_) there is only one crack propagating along the central line of the shear band (the lower scheme in the Fig. 5a). Transition to the region III is accompanied by backward shift of crack position to the “limiting” position. This behaviour is caused by changes in the degree of inhomogeneity of effective mean stress across the shear band with changing *A*_*xy*_. For *A*_*xy*_ several orders of magnitude larger than *A*_*crit*1_ the distribution of mean stress in the central part of the shear band is almost homogeneous, while the zone of maximum stress gradient is located relatively close to blocks (the cracks nucleate in this zone). For values of *A*_*xy*_ belonging to the region II (lower than *A*_*crit*1_), a narrow zone of lower effective mean stress forms in the centre of the shear band (Fig. 3b). This zone determines the position of the crack in the centre of the shear band. At *A*_*xy*_ < *A*_*crit*2_ the new factor appears, which leads to backward shift of the crack position. This factor is nonzero pore pressure in the shear band during the entire course of deformation. In the samples with *A*_*xy*_ lying in the region III this factor determines the profile of effective mean stress distribution across the shear band with maximum absolute value in the centre and the zone of maximum stress gradient located relatively close to the boundaries with the blocks.

## Discussion

The derived nonlinear and non-monotonic character of the dependence of the shear strength of mechanically constrained shear band on the parameter *A*_*xy*_ as well as the explanation of the reasons for this dependence are well supported by the results of recent experimental studies on triaxial deformation of different sandstone^31^ and edifice rock^40^ samples as well as on cone penetration tests in consolidated silts^59–62^. In particular, Duda and Renner^31^ showed in their detail study that non-monotonic dependence of the strength of permeable fluid-saturated samples on axial strain rate under constant value of nominal effective confining pressure is determined by a change in the internal drainage state. They concluded that effective drainage during deformation is determined by the interaction between the mechanical (deformation of the skeleton) and hydraulic (fluid redistribution) evolution. An important issue supported by issues of other cited experimental papers is that “bulk hydraulic properties rather than local inhomogeneities control the condition of drainage”^31^. Duda and Renner^31^ proposed an approximate expression to estimate critical strain rate corresponding to the local minimum of strength (the conditional point of transition from internal drained to undrained conditions):

4 $${\dot{{\rm{\varepsilon }}}}_{crit} \sim \frac{{{\rm{\Delta }}{\rm{\varepsilon }}}_{inelast}k}{s{\rm{\eta }}{l}^{2}},$$

where Δε_*inelast*_ denotes an interval of axial strains associated with significant volume changes, *s* is specific storage capacity, *l* is a characteristic length of fluid redistribution. Considering ${\dot{{\rm{\varepsilon }}}}_{crit}$ as an analogue of *A*_*crit*1_, we can estimate the values of the parameter *A*_*crit*1_ for sandstones studied by Duda and Renner^31^. Substituting the expression (4) for ${\dot{{\rm{\varepsilon }}}}_{crit}$ into the expression (3) for *A*_*xy*_ and applying the values of Δε_*inelast*_ and *s* for three experimentally studied sandstones we obtain an estimation of ${A}_{xy} \sim {{\rm{\Delta }}{\rm{\varepsilon }}}_{inelast}/s \sim {10}^{-1}\,\mbox{--}\,{10}^{0}\,{\rm{GPa}}$. This estimation corresponds within the order of magnitude to the numerically derived value *A*_*crit*1_ for the considered shear band. The all above said confirms our idea that non-monotonic profile of the dependence of strength on the parameter *A*_*xy*_ characterizing the ratio of strain rate to fluid flow rate is general for fluid- saturated rock samples and fragments of rock massifs where fracture has a localized pattern in the form of shear band formation or activation.

We have to emphasize that the key factor determining non-monotonic variation of the shear strength of a dilatant shear band at varying ratios of strain rate to fluid flow rate is mechanical constraint by the surrounding bulk of massif. Mechanical constraint in combination with dilation of the shear band leads to increasing compression of adjacent regions of rock and consequently to increase of effective stress. The rate of increase of effective stress determines the normal elastic stiffness of the surrounding massif. As shown in Fig. 2b, the rate of increase of effective stress, and consequently the elastic stiffness, nonmonotonously change with decreasing ratio of strain rate to fluid flow rate, and this change determines the shear strength variation.

The curve shown in Fig. 2b has three characteristic regions. In each region the change of strength is monotonous, which implies the presence of a dominant mechanism, which determines the direction of the change. When passing into a different region, both the dominant mechanism and the direction of the trend are changed.

In region I the dominating mechanism lies in the decrease of the linear dimensions of the blocks due to fluid outflow to the shear band (poroelastic contraction). Decrease in the value of *A*_*xy*_ is accompanied by the inflow of a large amount of fluid into the shear band and hence by reduction of the constraint imposed on the shear band by the compressed blocks (effective normal stiffness of the blocks decreases). This mechanism determines the decrease of the shear strength in region I as *A*_*xy*_ decreases.

In region II the trend-determining mechanism is tied to the increase of the dilation rate of the shear band with decreasing value of *A*_*xy*_ due to slowing of pore pressure reduction and maintaining nonzero pore pressure during most of the shear process. This mechanism provides an increase in the absolute value of effective mean stress in the sample and hence increase in the strength of the shear band in region II as *A*_*xy*_ decreases.

In region III the trend-determining mechanism is linked to the fact that pore pressure in the shear band remains non-zero during the entire shear process. In this region pore pressure in the shear band is higher in the samples characterized by lower values of A_xy_. Because of this fact an absolute value of effective mean stress in a shear band is also lower in the samples with lower A_xy_. Decrease of an absolute value of effective mean stress leads to gradual decrease in the shear strength down to the absolute minimum at *A*_*xy*_ → 0.

**Figure 6:**
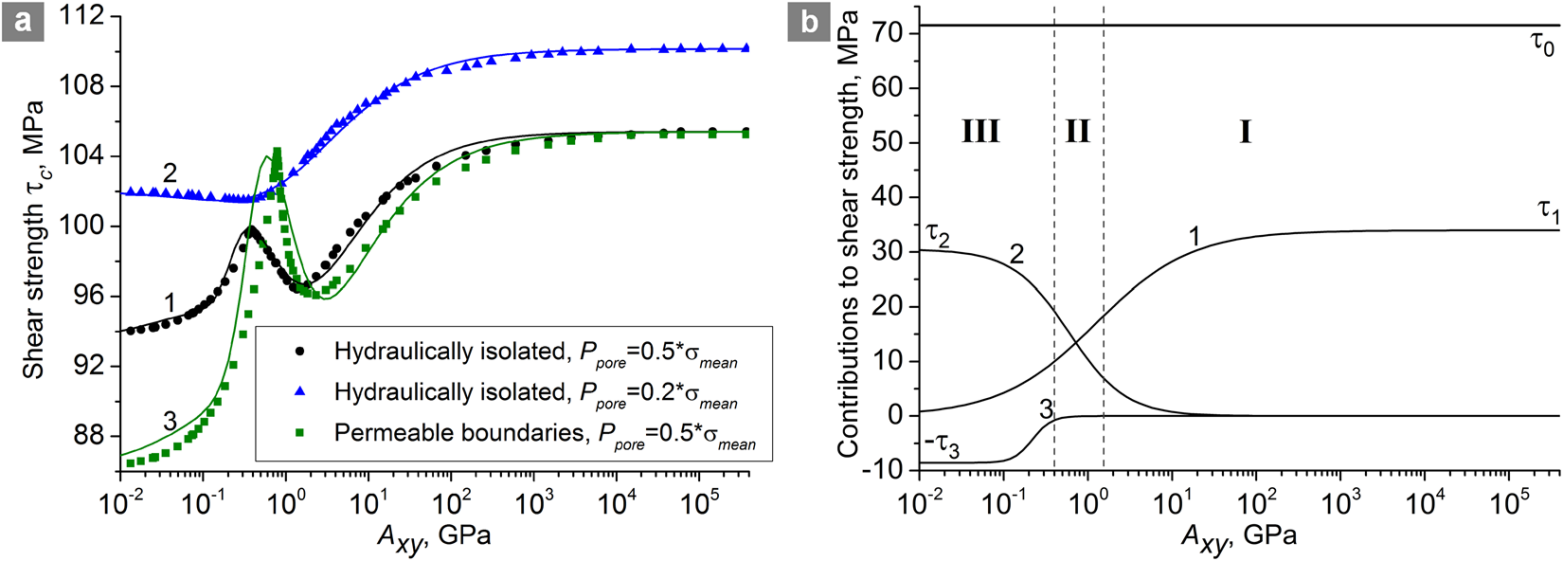
(**a**) Numerically derived sets of points τ_*c*_(*A*_*xy*_) approximated by Eq. (5) for hydraulically isolated samples at ${P}_{pore}^{0}=0.5|{\sigma }_{mean}^{0}|=17.5\,{\rm{MPa}}$ (curve 1), hydraulically isolated samples at ${P}_{pore}^{0}=0.2|{\sigma }_{mean}^{0}|=7\,{\rm{MPa}}$ (curve 2), and samples with perfectly permeable boundaries at ${P}_{pore}^{0}=0.5|{\sigma }_{mean}^{0}|=17\,{\rm{MPa}}$ (curve 3). In all examples ${\sigma }_{mean}^{0}=-35\,{\rm{MPa}}$, *h*/*H* = 0.05. (**b**) Examples of contributions to the shear strength τ_*c*_ of the shear band (regions I–III correspond to those in Fig. 2b). The presented contributions are used to approximate the set of points shown in Fig. 2b (their superposition is curve 1 in Fig. 6a). Lower *A*_*xy*_ imply faster fluid flow or lower strain rate, higher *A*_*xy*_ imply slower fluid flow or higher strain rate.

The described three parts of the curve τ_*c*_(*A*_*xy*_) have sigmoid profiles, which is the result of the competition between shear band dilatancy and poroelastic contraction of blocks due to fluid outflow. Analysis of the obtained result allowed to formulate the following general dependence of the shear strength of constrained shear band zones on the parameter *A*_*xy*_. The dependence is expressed as a sum of a constant and three sigmoid contributions (curve 1 in Fig. 6a):

5 $${{\rm{\tau }}}_{c}={{\rm{\tau }}}_{0}+\frac{{{\rm{\tau }}}_{1}}{1+{({c}_{1}{A}_{xy})}^{-{p}_{1}}}+\frac{{{\rm{\tau }}}_{2}}{1+{({c}_{2}{A}_{xy})}^{{p}_{2}}}-\frac{{{\rm{\tau }}}_{3}}{1+{({c}_{3}{A}_{xy})}^{{p}_{3}}},$$

where τ_1_, τ_2_ and τ_3_ are the amplitudes of contributions of the three above-mentioned mechanisms to the shear strength, *c*_1_, *c*_2_ and *c*_3_ are the inverse positions of the sigmoid midpoints, the exponents *p*_1_, *p*_2_ and *p*_3_ determine the steepness of the sigmoid functions and τ_0_ is a constant contribution independent of the fluid flow dynamics.

The amplitudes of the contributions have the following physical meanings. The constant contribution τ_0_ is the strength of the shear band in the absence of fluid flow (or under high strain rate) at a constant value of normal force σ_*N*_ applied to the upper and lower sample faces during the entire course of shear. Note that the dilation of the shear band doesn’t lead to increase in mean stress in the blocks under the given loading conditions. Another three contributions are concerned with squeezing of the blocks due to shear band dilation in a mechanically constrained sample.

The first sigmoid contribution dominating in region I is determined by a descending dependence of block’s normal stiffness on *A*_*xy*_ under constrained conditions. A value of τ_1_ can be experimentally estimated as a value of shear band strength at *A*_*xy*_ → ∞ under mechanically constrained sample faces minus τ_0_: ${{\rm{\tau }}}_{1}={{\rm{\tau }}}_{c}({A}_{xy}\to \infty )-{{\rm{\tau }}}_{0}$.

The third contribution is determined by the influence of non-zero pore pressure in the shear band on effective mean stress. A value of τ_3_ can be obtained from a constrained shear test at *A*_*xy*_ → 0, measuring pore pressure in the shear band. Special numerical analysis at different ${P}_{pore}^{0}$ and the fixed value of ${{\rm{\sigma }}}_{mean}^{0}=-35$ MPa has shown that the amplitude τ_3_ is proportional to the value of pore pressure ${P}_{pore}^{\max }$ in “fully drained” state of the shear band (*A*_*xy*_ → 0) at the moment of failure. This linear dependence is in good quantitative agreement with the analytical estimate derived from failure criterion (2): ${{\rm{\tau }}}_{3}=0.5\sqrt{3}(\lambda -1){P}_{pore}^{\max }$ (see Supplementary Materials).

The second sigmoid contribution is determined by the dependence of the dilation rate of a shear band material on the magnitude of pore pressure. Having experimentally estimated a value of τ_3_, it is possible to calculate τ_2_ in the following way: ${{\rm{\tau }}}_{2}={{\rm{\tau }}}_{c}({A}_{xy}\to 0)-{{\rm{\tau }}}_{0}-{{\rm{\tau }}}_{3}$.

Another six parameters can be estimated on the basis of fitting the curve to experimental data on the shear strength in intermediate range of *A*_*xy*_. More promising way is a development of an analytical approach allowing estimation of these parameters based on material properties. This is a subject of further research. However, we should mention that analytical estimation ${A}_{crit1} \sim {\rm{\Delta }}{\varepsilon }_{inelast}/s$ makes determining of parameters of the first and the second sigmoidal contributions into Eq. (5) much easier.

The specific parameter values of the Eq. (5) depend on mechanical characteristics of the skeleton of shear band and blocks, bulk modulus of fluid, the *h*/*H* ratio, initial mean stress ${\sigma }_{mean}^{0}$, fluid content in the blocks and hydrological conditions. An example of constant and sigmoid contributions to shear band strength for the particular case shown in Fig. 2b are drawn in Fig. 6b.

Figure 6a shows examples of the τ_*c*_(*A*_*xy*_) dependences for different pore pressure values and hydrological boundary conditions (curves 1–3) approximated by Eq. (5). Comparison of the curves 1 and 2 shows the determining role of fluid content for the non-monotonic pattern of variation of τ_*c*_. Decrease in initial pore pressure leads to “degeneration” of the curve to the simple sigmoid function (τ_3_ approaches zero, τ_1_ and τ_2_ decrease) and then to trivial τ_*c*_ = *const* at ${P}_{pore}^{0}\to 0$. The strength of a shear band also strongly depends on the permeability of the host massif enclosing the shear band and adjacent regions of blocks. At a relatively high host massif permeability (curve 3, Fig. 6a) modelled by means of “perfectly permeable” boundary conditions, the amplitudes τ_2_ and τ_3_ increase, compared with the same parameters for a hydraulically isolated system (curve 1, Fig. 6a), while the parameters of the constant and first contributions remain unchanged. This leads to a sharp increase in the nonmonotonicity of the curve including a pronounced increase in the value of the local maximum and considerably lower value of the minimum at *A*_*xy*_ → 0.

**Figure 7:**
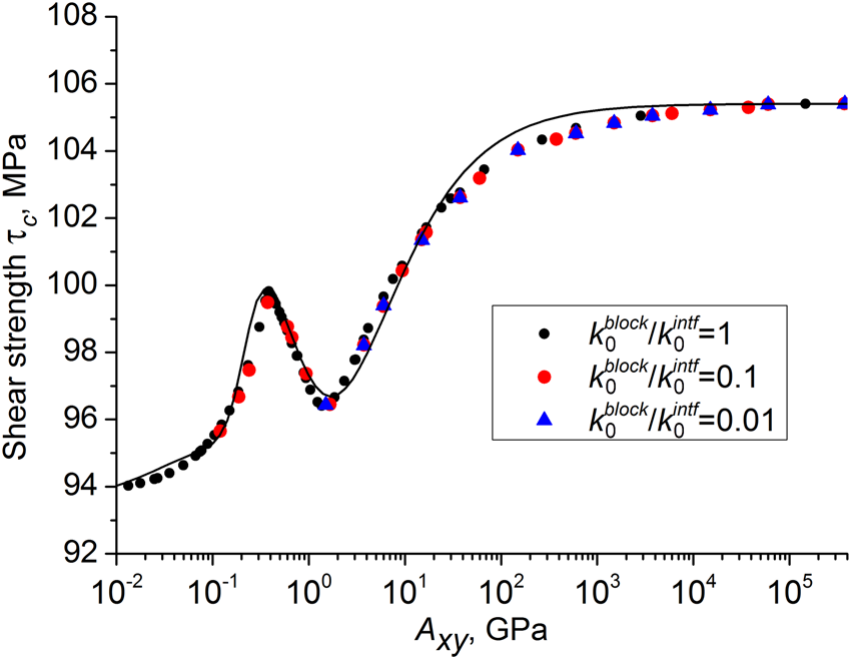
Numerically derived sets of points τ_*c*_(*A*_*xy*_) approximated by Eq. (5) for hydraulically isolated samples (*h*/*H* = 0.05, ${P}_{pore}^{0}=0.5|{{\rm{\sigma }}}_{mean}^{0}|=17\,{\rm{MPa}}$) with different values of permeability of blocks (${k}_{0}^{block}$) and shear band (${k}_{0}^{intf}$). The parameter *A*_*xy*_ is calculated using ${k}_{0}^{block}$. The top and the bottom faces of the samples are fixed in vertical direction. Lower *A*_*xy*_ imply faster fluid flow or lower strain rate, higher *A*_*xy*_ imply slower fluid flow or higher strain rate.

Finally, we stress that in this study we assumed the permeability of the blocks to be same as shear band permeability, which is reminiscent of the situation in the central zones of lithified fault segments. At the same time, it is a widespread situation that the permeability of the shear band is higher than permeability of surrounding blocks. In particular, permeability of unconsolidated shear band may be several orders of magnitude higher than block permeability. Separate analysis of such systems has shown that the form of the relationship (5) and the values of its parameters do not change if the combination *A*_*xy*_ is defined as follows: ${A}_{xy}={\dot{{\rm{\varepsilon }}}}_{xy}{\rm{\eta }}{(H+h)}^{2}/{k}_{0}^{block}$, where ${k}_{0}^{block}$ is block permeability (Fig. 7). So, the dependence τ_*c*_(*A*_*xy*_) is general for shear band zones, where block permeability does not exceed shear band permeability (at the same ϕ_0_). This allows to state that it is the permeability of the blocks that controls the shear band strength in rock massifs, all other factors being equal.

The shear band strength is directly connected with the length of the stable stage of pervasive shear (Fig. 2a). The results of this study show that the length of this stage can change (and potentially can be managed) as a result of changes in shear rate, fluid viscosity and permeability of the shear band zone and surrounding massif. Depending on the magnitude and sign of change of the parameters that determine the instantaneous value of *A*_*xy*_, this change can be positive (elongation) or negative (shortening). The latter may be especially important for control of the point of transition of the regime of shear of consolidated fault segments from stick to dynamic slip.

## Methods

We numerically simulated constrained shear of a model shear band using one of the representatives of the group of discrete element methods (DEM), namely the explicit method of simply deformable elements. The term “explicit” means that evolution of an ensemble of interacting discrete elements is defined by a numerical solution of the system of classical equations of motion using an explicit numerical scheme (the velocity Verlet algorithm in our case)^48^. We use the well-known approximation of equivalent discs in the 2D problem statement for interpreting the element shape when numerically solving equations of motion (this kind of implementation of DEM is often called a distinct element method)^47,48^. This approximation allows to use simplified (Newton-Euler) equations of motion and to divide the interaction between two discrete elements into two independent components: central (oriented along the line connecting the centres of mass of the elements) and tangential (in the plane transverse to the mentioned line). In the simply deformable element approximation the state of a discrete element is determined by the average stress tensor σ_αβ_ and average strain tensor ε_αβ_, which are calculated through interaction forces, pair overlaps and relative shear displacements in the interacting pairs of the element with its neighbours^47,49^. One important feature of our implementation of the simply deformable DEM is the use of the original many-body formulation of the element-element interaction^49,63^. It allowed us to implement coupled models of poroelasticity, poroplasticity and fluid affected failure within the DEM. Detailed description of the features of the numerical method and model implementation can be found in the papers^49,50^.

The problem of modelling the mechanical behaviour of permeable fluid-saturated material by an ensemble of interacting discrete elements is divided into two subproblems: (1) description of the mechanical behaviour of the enclosing solid skeleton with interstitial fluid; (2) description of fluid transfer in the filtration volume (system of connected channels, pores and microcracks) of the solid. In the considered model we assume the characteristic thickness of pores, channels and microcracks to be smaller than the discrete element size. Therefore, the discrete elements are treated as porous and permeable. The influence of pore fluid in the volume of the element on its mechanical properties and response is described implicitly with use of the models of poroelasticity and poroplasticity.

We used Biot’s linear model of poroelasticity to describe the mutual relation between average stresses and strains and fluid pore pressure in the volume of the discrete element. The formulation of the constitutive equation for a fluid-filled porous isotropic elastic solid is used^54^:

6 $${{\rm{\sigma }}}_{{\rm{\alpha }}{\rm{\beta }}}=2G({{\rm{\varepsilon }}}_{{\rm{\alpha }}{\rm{\beta }}}-\frac{a{P}_{pore}}{K}{{\rm{\delta }}}_{{\rm{\alpha }}{\rm{\beta }}})+(1-\frac{2G}{K}){{\rm{\sigma }}}_{mean}{{\rm{\delta }}}_{{\rm{\alpha }}{\rm{\beta }}},$$

where α, β = *x*, *y*, *z*; *G* and *K* are the shear and bulk elastic moduli of the material of the element; δ_αβ_ is the Kronecker delta; *a* is the poroelastic constant proportional to the ratio of bulk moduli of porous and nonporous material, *P*_*pore*_ is the average value of pore pressure in the volume of the element.

The behavior of the material of the discrete element beyond the yield stress is simulated using Nikolaevsky’s rock plasticity model with a nonassociated flow rule and modified Mises–Schleicher yield criterion (1), which handles effective mean stress in the volume of the discrete element ${{\rm{\sigma }}}_{mean}^{eff}={{\rm{\sigma }}}_{mean}+{P}_{pore}$ instead of conventional mean stress in solid skeleton σ_*mean*_. A feature of Nikolaevsky’s model is that it postulates a linear dependence between the rates of the bulk and shear plastic strain components with a proportionality factor Λ, termed the dilatancy factor. Nikolaevsky’s model is implemented within the DEM formalism with use of the radial return algorithm of Wilkins. According to the algorithm, a numerical solution of an elastic-plastic problem at each step of integrating the equations of motion consists of: (1) incremental solution of an elastic problem and (2) subsequent reduction of the average stress tensor σ_αβ_ in the volume of the discrete element to the yield surface *Y* if the inequality ${\rm{\Phi }}={{\rm{\beta }}{\rm{\sigma }}}_{mean}^{eff}+{{\rm{\sigma }}}_{eq}/\sqrt{3} > Y$ holds true^49^:

7 $${{\rm{\sigma }}^{\prime} }_{{\rm{\alpha }}{\rm{\beta }}}=({{\rm{\sigma }}}_{{\rm{\alpha }}{\rm{\beta }}}-{{\rm{\sigma }}}_{mean}{{\rm{\delta }}}_{{\rm{\alpha }}{\rm{\beta }}})M+({{\rm{\sigma }}}_{mean}-N){{\rm{\delta }}}_{{\rm{\alpha }}{\rm{\beta }}},$$

where ${{\rm{\sigma }}^{\prime} }_{{\rm{\alpha }}{\rm{\beta }}}$ are the reduced values of the components of the average stress tensor in the volume of the discrete element, $M=1-(\sqrt{3}/{{\rm{\sigma }}}_{eq})(3G({\rm{\Phi }}-Y)/(K{\rm{\Lambda }}{\rm{\beta }}+3G))$ is the reduction factor of stress deviators, *N* = *K*Λ(Φ − *Y*/(*K*Λβ + *G*)) is the correction of the mean stress value calculated in an elastic approximation. Nikolaevsky’s plasticity model of brittle materials is a typical representative of nonassociated flow models. Its main limitation is the neglect of possible pore collapse. Therefore, this model is typically applied to brittle materials with relatively low initial porosity (less than 15–20%) at mean stresses below the brittle-ductile transition threshold^64,65^.

The rheological properties of the dry material of a discrete element are assigned through the dependence *Y*(*ε*_*ms*_), where ${{\rm{\varepsilon }}}_{ms}={{\rm{\varepsilon }}}_{mean}K{\rm{\beta }}/3G+{{\rm{\varepsilon }}}_{eq}/\sqrt{3}$ is a combination of mean strain ε_*mean*_ and equivalent strain ε_*eq*_ (here we imply total values of strains including both elastic and irreversible parts)^49^. The strain parameter ε_*ms*_ can be conventionally called “modified Mises-Schleicher strain parameter”). The form of parameter ε_*ms*_ ensures the equality Φ/ε_*ms*_ = 3*G* within the region of elastic behavior (ε_*ms*_ is an analog of equivalent strain in the conventional models of plasticity of metals with von Mises yield criterion). The dependence *Y*(ε_*ms*_) for dry material (*P*_*pore*_ = 0) is easily calculated from available or assigned stress-strain curves under shear or uniaxial compression. The strain hardening modulus Π is a derivative Π = *d*Φ/*d*ε_*ms*_.

The modified failure criterion of Drucker and Prager (2), which takes into account local pore fluid pressure is used as the criterion of bond failure (linked pair → unlinked pair) in pairs of interacting fluid-saturated discrete elements^49^.

Note that the yield and failure criteria (1–2) are generalizations (smoothing) of the Mohr-Coulomb yield and failure criteria, which are widely used in rock and fault mechanics^4,7,24,38,57^. The main distinction of criteria (1–2) is that they take into account the intermediate principal stress along with the major and minor principal stresses. Typically the estimates of yield stress and strength by (1–2) are quite close to Mohr-Coulomb criteria, and main limitations and working ranges of (1–2) as well as sensitivity of the results to the values of criterion parameters are similar for both kinds of criteria.

The filtration volume (also called the open porosity) Ω_*pore*_ of the discrete element is considered to be the sum of two components: the volume of the initial system of connected pores, cracks and channels (Ω_*pore*,*el*_) and the volume of new pores (Ω_*pore*,*pl*_) formed due to material dilatancy during inelastic deformation (i.e., due to irreversible opening of microscopic discontinuities). Reversible changes of Ω_*pore*,*el*_ during elastic deformation of porous material are described within the framework of Biot’s linear model of poroelasticity^54^:

8 $${{\rm{\Omega }}}_{pore.el}={{\rm{\Omega }}}_{0}[{{\rm{\varphi }}}_{0}+\frac{3a{{\rm{\sigma }}}_{mean}}{K}+\frac{3{P}_{pore}}{K}(1-a(1+{\rm{\varphi }}))],$$

where Ω_0_ is the initial volume of the element, ϕ_0_ is the initial porosity. The irreversible change of pore volume Ω_*pore*,*pl*_ due to material dilatancy is expressed through the plastic component of bulk strain of the discrete element θ_*plast*_:

9 $${{\rm{\Omega }}}_{pore,pl}={{\rm{\Omega }}}_{0}{{\rm{\theta }}}_{plast}={{\rm{\Omega }}}_{0}({\rm{\theta }}-{{\rm{\theta }}}_{elast})={{\rm{\Omega }}}_{0}[({{\rm{\varepsilon }}}_{xx}+{{\rm{\varepsilon }}}_{yy}+{{\rm{\varepsilon }}}_{zz})-3({{\rm{\sigma }}}_{mean}+a{P}_{pore})/K],$$

where θ and θ_*elast*_ are the total bulk strain of the element and its reversible (elastic) part, ε_αα_ are diagonal components of the average strain tensor in the element volume.

Permeability *k* of the material of a discrete element is treated as a function of porosity ϕ = Ω_*pore*_/Ω_0_ and the so called “characteristic diameter” of filtration channels *d*_*ch*_^49,66^:

10 $$k={\rm{\varphi }}{d}_{ch}^{2}.$$

In the general case the parameter *d*_*ch*_ is not the average transverse size of discontinuities, but the size of the narrowest channels in the crack-pore space, which determine the rate of fluid flow through the volume of the discrete element.

We considered interstitial fluid to be compressible and used the classical equation of state^66^: ${{\rm{P}}}_{pore}=$ ${K}_{fl}({{\rm{\rho }}}_{pore}/{{\rm{\rho }}}_{0}-1)$, where *K*_*fl*_ is the bulk modulus of interstitial fluid, ρ_*pore*_ = *m*_*fl*_/Ω_*pore*_ is fluid density in the pore space of the element (*m*_*fl*_ is mass of fluid in pore space), ρ_0_ is the equilibrium value of fluid density under atmospheric conditions. We assume *P*_*pore*_ = 0 if ρ_*pore*_ ≤ ρ_0_. The fluid pressure gradient is assumed to be a driving force of filtration. We used the classical equation of fluid mass transfer in the “micropore” space^66^:

11 $${\rm{\varphi }}\frac{{\rm{\partial }}{{\rm{\rho }}}_{pore}}{{\rm{\partial }}t}={K}_{fl}\nabla [\frac{k}{{\rm{\eta }}}\nabla {{\rm{\rho }}}_{pore}].$$

This equation is numerically solved using the Euler method on a grid formed by an ensemble of interacting discrete elements. Here grid nodes are the centres of mass of discrete elements. There is no mass transfer between grid nodes in which ρ_*pore*_ ≤ ρ_0_.

The described model was verified in a series of numerical tests including uniaxial compression of samples of dry model materials with different values of the dilatancy factor Λ, fluid filtration through a thin layer of permeable material and fluid discharge from a sample under rapid uniaxial compression (without failure). Verification results are shown in Supplementary Materials.

### Data availability

The datasets generated during and/or analyzed during the current study are available from the corresponding author on reasonable request.

## Electronic supplementary material

Supplementary Materials
