# Supplementary material for: Strength of shear bands in fluid-saturated rocks: a nonlinear effect of competition between dilation and fluid flow
Source: Sci Rep. 2018 Jan 23;8:1428. doi: 10.1038/s41598-018-19843-8 (PMC5780515; doi:10.1038/s41598-018-19843-8)
Supplement: Supplementary file 1 — Supplementary Materials [file 41598_2018_19843_MOESM1_ESM.doc]

Supplementary Materials for

# Strength of shear bands in fluid-saturated rocks: a nonlinear effect of competition between dilation and fluid flow

Evgeny V. Shilko^1,2,*^, Andrey V. Dimaki^1^, and Sergey G. Psakhie^1,3^

^1^Institute of Strength Physics and Materials Science SB RAS, Laboratory of Computer-Aided Design of Materials, Tomsk, 634055, Russia

^2^Tomsk State University, Faculty of Physics, Tomsk, 634050, Russia

^3^Tomsk Polytechnic University, Institute of High Technology Physics, Tomsk, 634050, Russia

^*^Corresponding author. E-mail: [shilko@ispms.tsc.ru](mailto:shilko@ispms.tsc.ru)

1. **Verification of the model of plasticity within the discrete element model**

Verification of the developed model of an elastic-plastic material shows that an ensemble of interacting discrete elements demonstrates macroscopically isotropic mechanical response even in the case of uniform packing of equal-size elements (this is an important problem when pair-wise interaction is used) [1]. A correctness and accuracy of the proposed model are illustrated using an example of uniaxial loading of 2D sample under the plane stress condition. The sample consists of about 6000 close-packed equal-size discrete elements (Supplementary Figure S1). The sample size is 6×10 mm, the size of an element is *d*=0.1 mm. The lower row of elements in the sample is fixed in vertical direction (the condition of zero vertical displacement). The upper row of elements moves in vertical direction with constant velocity in a quasi-static regime of deformation. Elements in both rows can move free in horizontal direction, that is an equivalent of σ*_xx_*=τ*_xy_*=0 boundary condition. A model of elastic-plastic material with piecewise linear hardening was considered. The parameters of elastic response of the material have the following values: Young modulus *E*=80 GPa, Poisson’s ratio μ=0.3, yield stress σ*_y_*=80 MPa.

| 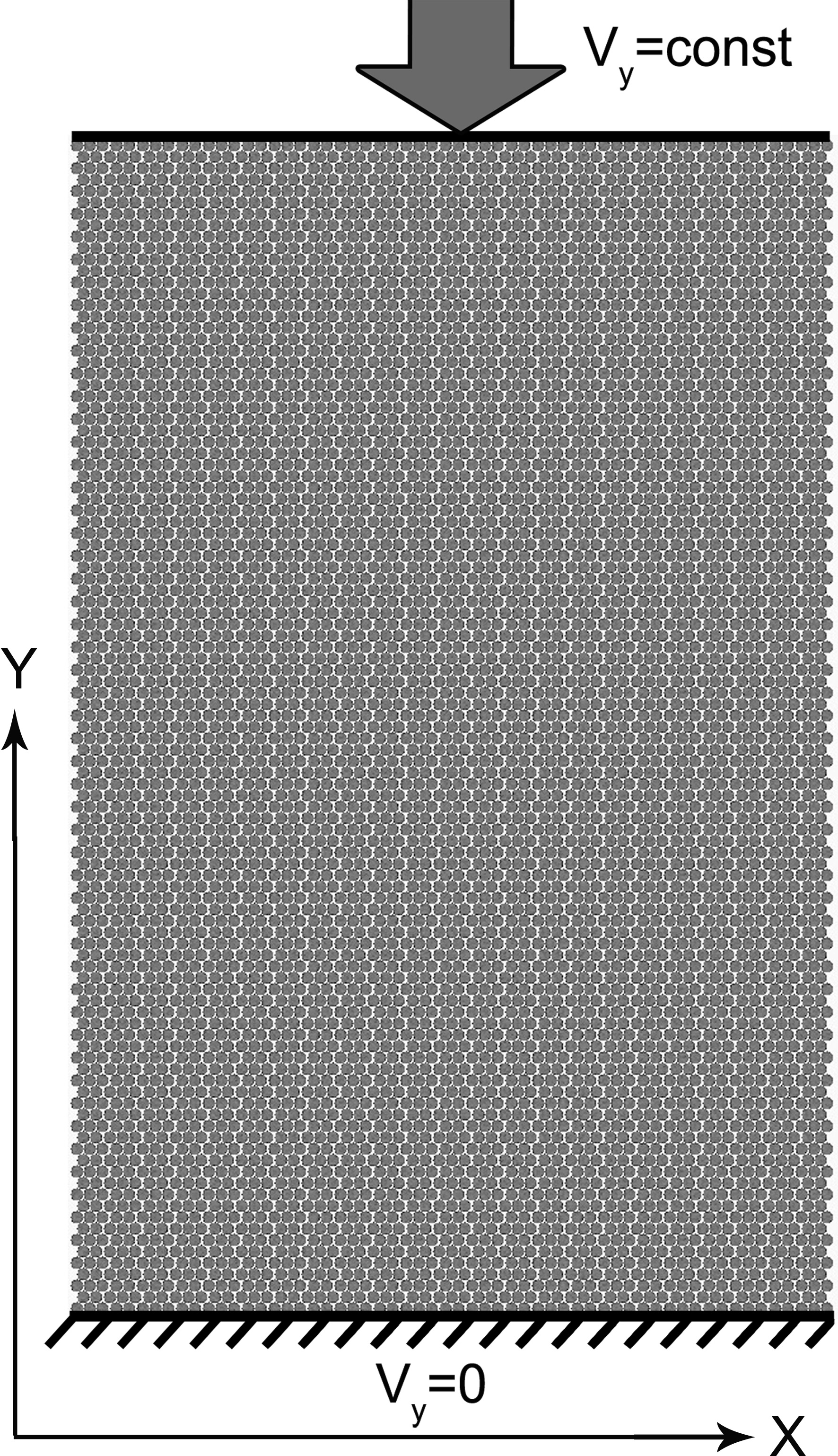 |
| --- |
| **Figure S1.** Structure of the 2D model setup and schematic representation of loading conditions. |

The implemented Nikolaevsky's model of non-associated plastic flow reproduces a model of metal plasticity with von Mises yield criterion under the condition of ω=Λ=0. This partial case is considered in the following test. Supplementary Figure S2 shows the specific value of reaction force (axial stress σ) and lateral expansion coefficient ν versus axial strain ε. These two quantities characterize a macroscopic response of an elastic-plastic sample under uniaxial compression. Coefficient of lateral expansion ν was determined as a ratio of lateral sample strain ε*_lateral_* to axial strain ε:

$\nu=\left|\frac{\varepsilon_{lateral}}{\varepsilon}\right|=\left|\frac{\left(W-W_{0}\right)/W_{0}}{\varepsilon}\right|$, (S.1)

where *W* is the current sample size in the transverse direction to the axis of loading, *W*_0_ is initial sample width. In the region of elastic deformation this coefficient has the meaning of Poisson’s ratio. Having an analytic dependence $\sigma_{yy}(\varepsilon_{yy})$ it is possible to obtain an equation for the coefficient of lateral expansion in the following obvious form:

$\nu=\frac{\varepsilon_{xx}}{\varepsilon_{yy}}$. (S.2)

Here ε*_xx_* consists of elastic ($\varepsilon_{xx}^{elast}$) and inelastic ($\varepsilon_{xx}^{inelast}$) parts:

$\varepsilon_{xx}=\varepsilon_{xx}^{elast}+\varepsilon_{xx}^{inelast}$.

Elastic contribution to ε*_xx_* is calculated on the basis of Hooke’s law:

$\varepsilon_{xx}^{elast}=-\nu_{0}\varepsilon_{yy}=-\frac{\nu_{0}}{E}\sigma_{yy}$,

where $\nu_{0}$ is Poisson’s ratio of the material. The expression for inelastic contribution to lateral expansion of the sample under uniaxial loading is derived from the expression for plastic strain rate in Nikolaevsky’s rock plasticity model [2]:

,

where $\lambda$ is a multiplier, which has dimension σ^-1^ (Pa^-1^) and equal to zero in the region of pure elastic response. Applying this expression for uniaxial loading ($\sigma_{mean}=\sigma_{yy}/3$, $\sigma_{eq}=\left|\sigma_{yy}\right|$, $Y=\beta\sigma_{yy}/3+\left|\sigma_{yy}\right|/\sqrt{3}$), we obtain the following expressions for diagonal components of plastic strain rate tensor:

,

,

which make possible to directly express “lateral” components of plastic strain rate tensor through “axial” component :

,

or:

$\varepsilon_{xx}^{inelast}=\varepsilon_{zz}^{inelast}=\left[\frac{\frac{\Lambda}{\sqrt{3}}-\frac{\mathrm{sgn}\left(\sigma_{yy}\right)}{2}}{\frac{\Lambda}{\sqrt{3}}+\frac{\mathrm{sgn}\left(\sigma_{yy}\right)}{2}}\right]\varepsilon_{yy}^{inelast}$.

Final expression for lateral strain of uniaxially loaded sample as a function of the axial stress and total axial strain takes the following form:

$\varepsilon_{xx}=-\frac{\nu_{0}}{E}\sigma_{yy}+\left[\frac{\frac{\Lambda}{\sqrt{3}}-\frac{\mathrm{sgn}(\sigma_{yy})}{2}}{\frac{\Lambda}{\sqrt{3}}+\frac{\mathrm{sgn}(\sigma_{yy})}{2}}\right]\left(\varepsilon_{yy}-\frac{\sigma_{yy}}{E}\right)$. (S.3)

Simulation results were compared with analytical solution (S.2)-(S.3) for the considered particular case of uniaxial compression of a homogeneous sample (see dash-dot curves in Supplementary Figure S2). There is good agreement between numerical and analytical solutions (the discrepancy is about 2%). Decrease of element diameter *d* under constant sample size leads to convergence of numerically obtained curves to analytical results.

| 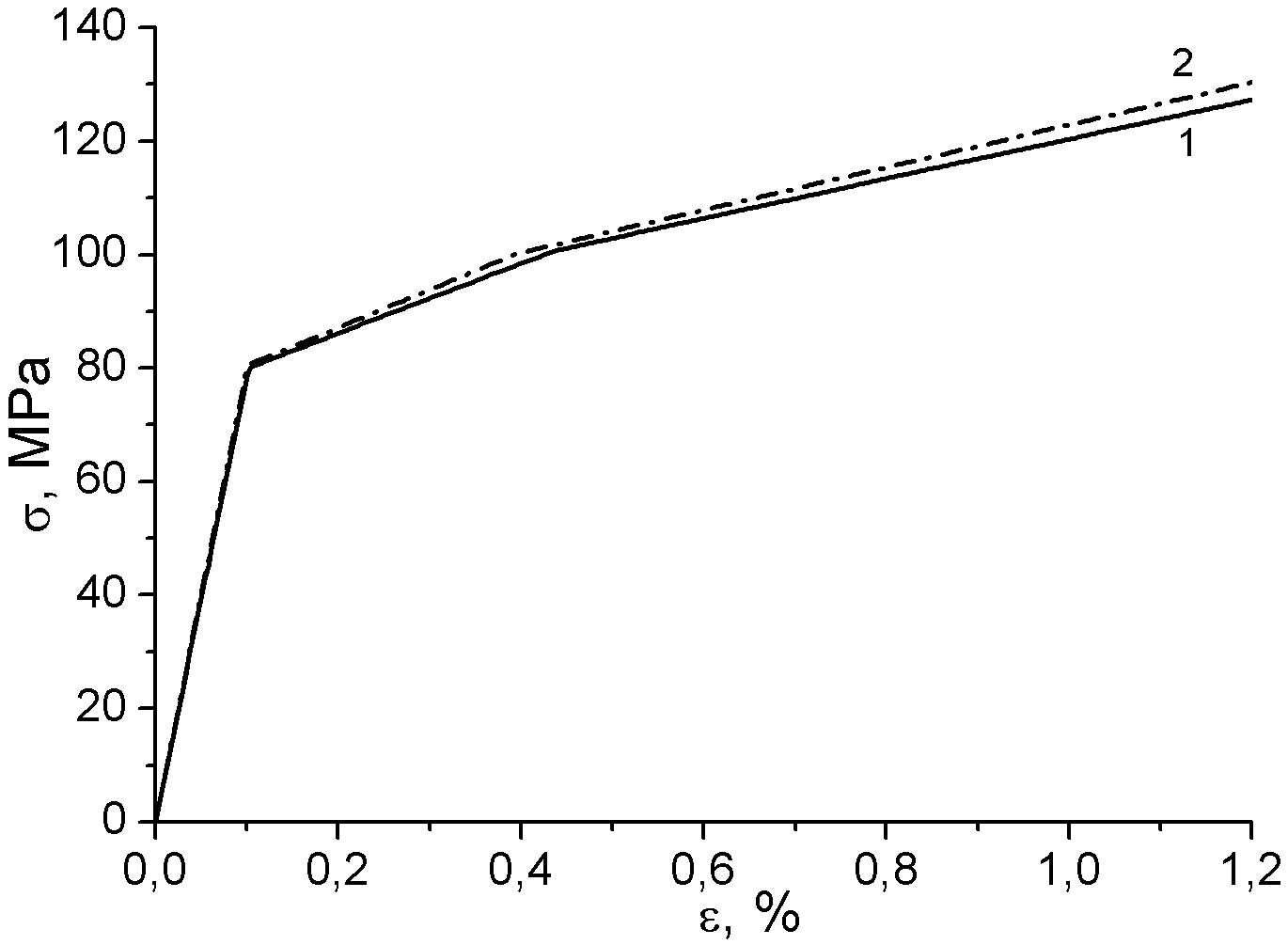 | 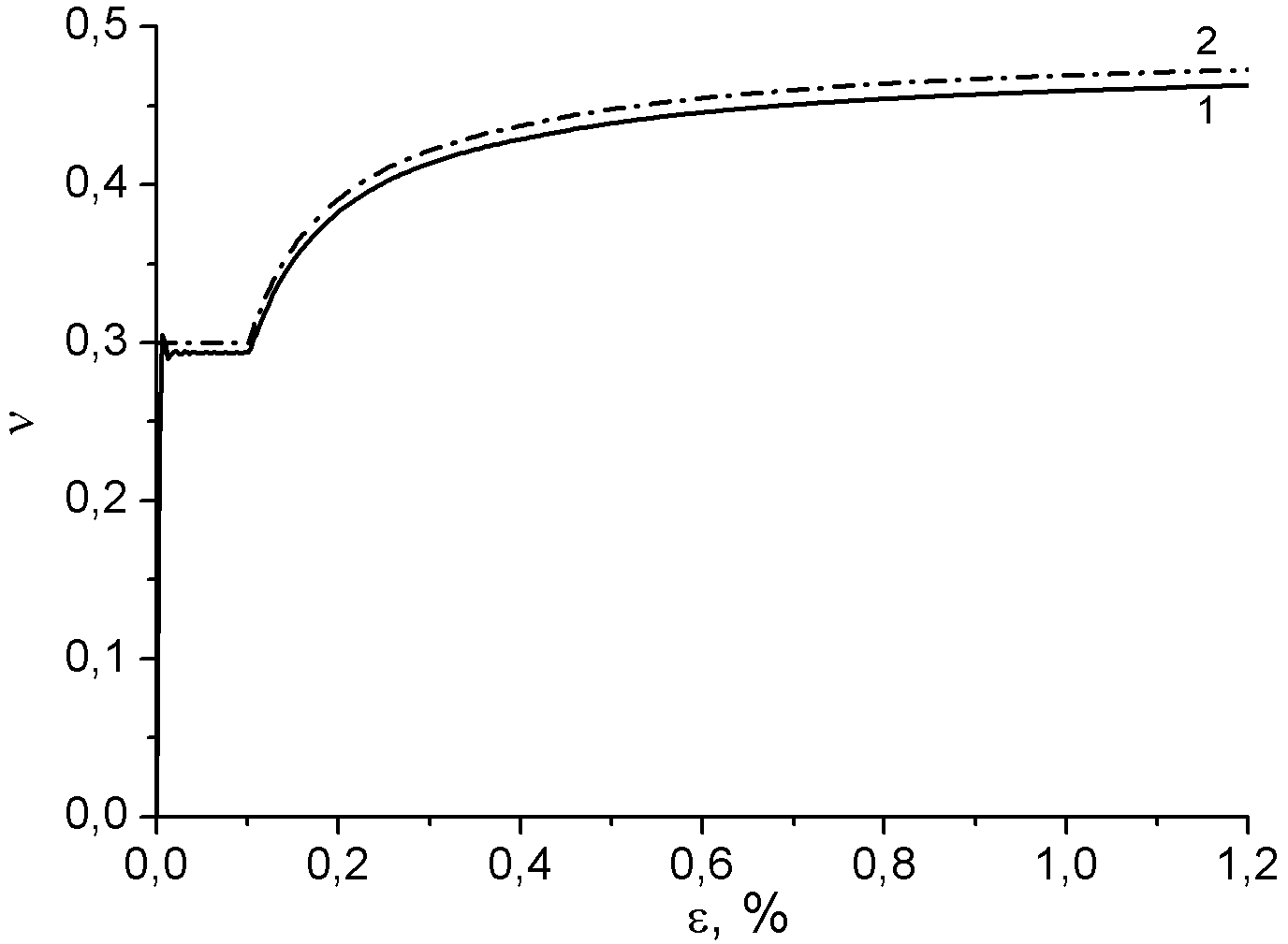 |
| --- | --- |
| a) | b) |
| **Figure S2.** Dependences of axial stress σ (a) and sample lateral expansion coefficient ν (b) on applied axial deformation ε under uniaxial compressive loading: 1 – simulation results; 2 – analytical solution. Absolute values of σ, ε and ν are plotted. Particular case of the model of plasticity of metals with von Mises yield criterion (ω=Λ=0) is considered. | |

Influence of parameters of Nikolaevsky’s model of plasticity on material behavior under uniaxial loading is shown in Supplementary Figure S3. There is a difference in material behavior under compression (curve 1) and tension (curve 2) in the case of non-zero internal friction coefficient ω=0.1 (the coefficient of dilation Λ=0 was used). Inelastic deformation under tension begins at lower applied stress, compared to compression. The results of numerical simulation of uniaxial compression and tension are compared with corresponding analytical solutions (dash-dot curves 1’ and 2’ respectively). As in the previous case, there is good agreement of corresponding numerical and analytical solutions (relative difference is less than 3%). Note that the difference between compressive and tensile yield stresses depends on the value of internal friction coefficient ω. In the particular case ω=0, that corresponds to von Mises plasticity model, dependences σ(ε) for tension and compression coincide, while their divergence increases as ω increases.

| 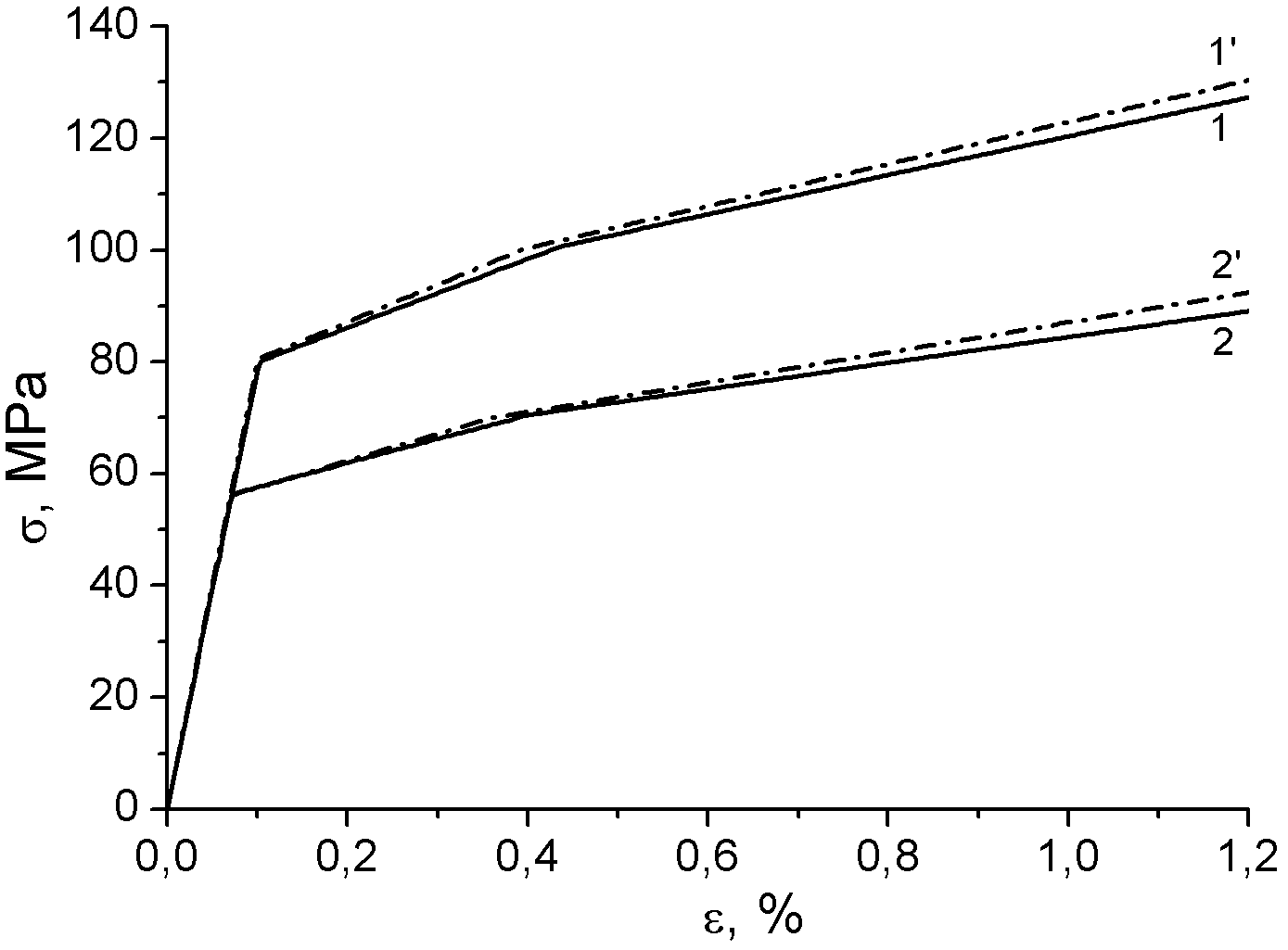 |
| --- |
| **Figure S3.** Dependences of axial stress σ on applied axial deformation ε under uniaxial compression (curve 1) and tension (curve 2). Absolute values of σ, ε and ν are plotted. Curves 1′ and 2′ show corresponding analytical solutions for curves 1 and 2. All curves were obtained for the material with ω=0.1 and Λ=0. |

Supplementary Figure S4 demonstrates dependences ν(ε) for three different values of Λ. The value of ω=0.1 was used in these calculations. Simulation results show that increase in the coefficient of dilatancy from 0 to 0.3 leads to increase in lateral expansion of material nearly twice. At the same time an influence of Λ on loading diagram is negligibly small in the case of uniaxial loading. The obtained numerical results for different values of Λ are in good agreement with corresponding analytical solutions (compare curves 1’-3’ and 1-3 respectively).

| 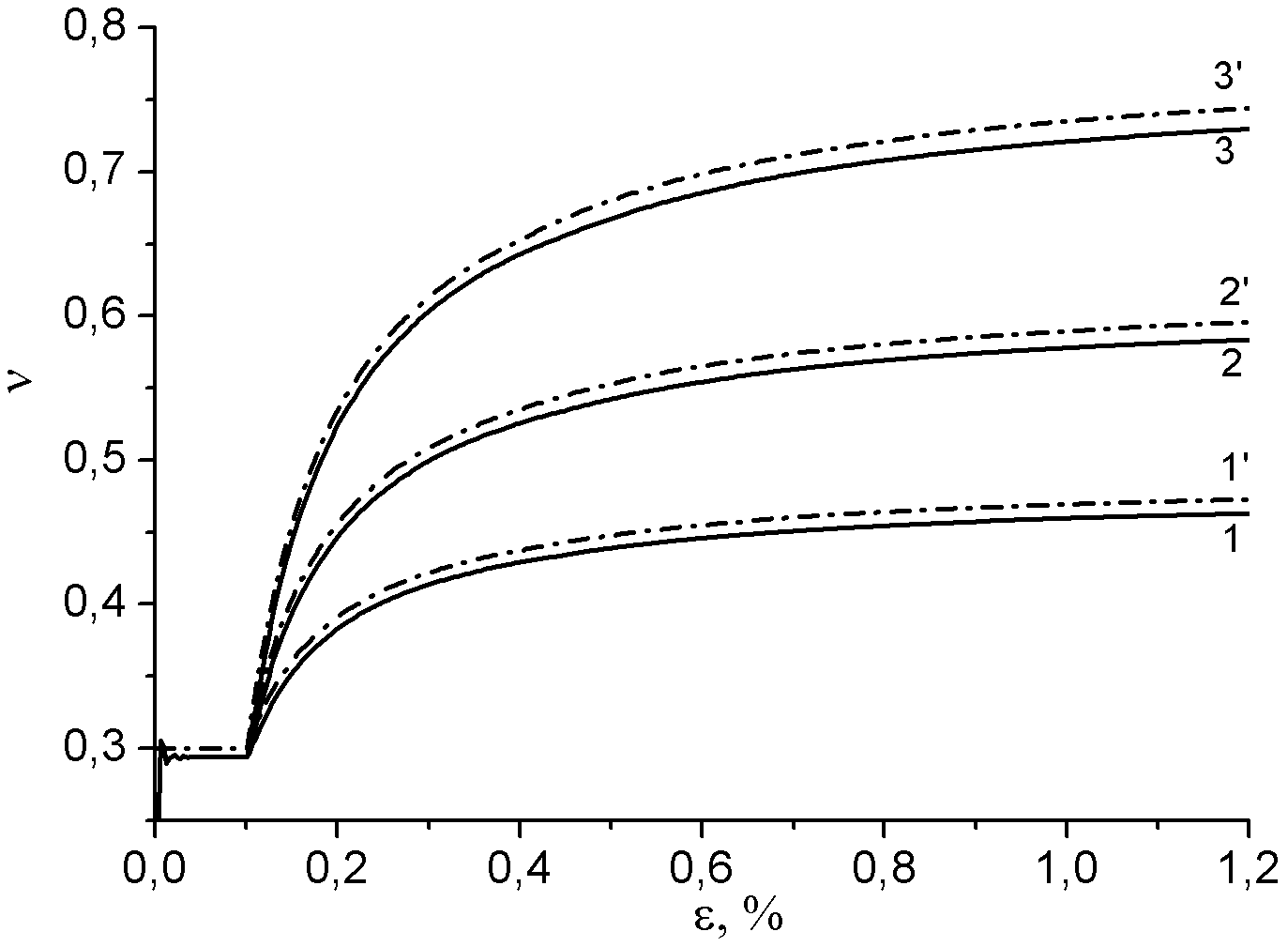 |
| --- |
| **Figure S4.** Dependences of sample lateral expansion coefficient ν on applied axial deformation ε under uniaxial compression. Different curves correspond to different values of the coefficient of material dilatancy Λ: 0 (curve 1); 0.15 (curve 2); 0.3 (curve 3). Absolute values of ν and ε are plotted. The same value ω=0.1 was used in all calculations. Curves 1′-3′ demonstrate corresponding analytical solutions for numerical curves 1-3. |

Good qualitative and quantitative agreement of the numerical results with analytical solutions shows the adequacy and correctness of the developed mathematical model of the interaction of simply deformable discrete elements for simulation of the mechanical response of elastic-plastic materials.

1. **Verification of the model of filtration**

The developed fully coupled model was verified in a series of numerical tests including: 1) fluid filtration through a thin layer of permeable material; 2) fluid discharge from a sample under rapid uniaxial compression [3]. Fluid filtration through a thin layer was modeled for a sample of porous elastic-brittle material of length $L=0.94$ m and width $W_{s}=0.0035$ m using the following initial

$$P\left(t=0\right)=0 \text{MPa}$$

and boundary conditions in the layer:

$$\begin{matrix} P\left(x=0\right)=0.43 \text{MPa} \\ P\left(x=L\right)=0 \text{MPa} \end{matrix}$$

The filtered fluid was water with viscosity of $\mu=10^{-3} \text{Pa}\cdot\text{sec}$ and the bulk modulus of $K_{liq}=2.2 \text{GPa}$. The initial microporosity of the sample was , its permeability was $k=10^{-15} \text{m}^{2}$. At the initial time micropores in the sample were assumed to be completely filled with water, at that pore fluid pressure was assumed to be zero. The schematic of the modeled sample is given in Supplementary Figure S5.

| 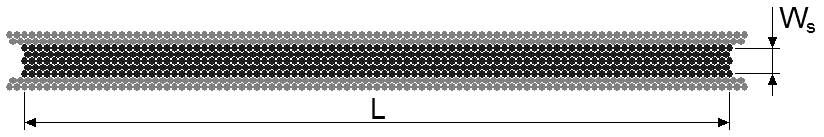 |
| --- |
| **Figure S5**. Schematic of a permeable sample for fluid filtration modeling. |

The simulation results were compared to the estimates obtained with the use of the following semi-analytical model. If fluid is assumed to be weakly compressible and pore deformation under the action of fluid is neglected, pressure distribution in a narrow permeable sample may be approximated by the series [4]:

. (S.4)

One can approximate the pressure distribution in the sample with required accuracy by means of calculation of a sufficient number of terms in the series (S.4),

In order to verify the model adequacy and to study how an explicit account for poroelasticity affects pressure distribution in the sample, fluid filtration was modeled in the following two approximations:

1. Porosity doesn’t depend on fluid pressure and stress state of the material: ;

2. Porosity depends on mean stress and pore pressure (poroelastic material).

In both cases, the numerically simulated pore fluid pressure profiles differ from estimate (S.4) by no more than 10% (Supplementary Figure S6). As it is seen from Supplementary Figure S6b, taking into account poroelasticity leads to a pore pressure decrease compared to the model in which the change of pore volume in a solid is not taken into account. This effect is observed even at relatively low pore pressure values. The obtained results demonstrate the necessity of taking into account the effect of poroelasticity in calculations of mechanical state of fluid-saturated samples subjected to high pore fluid pressure or high mechanical loads.

| 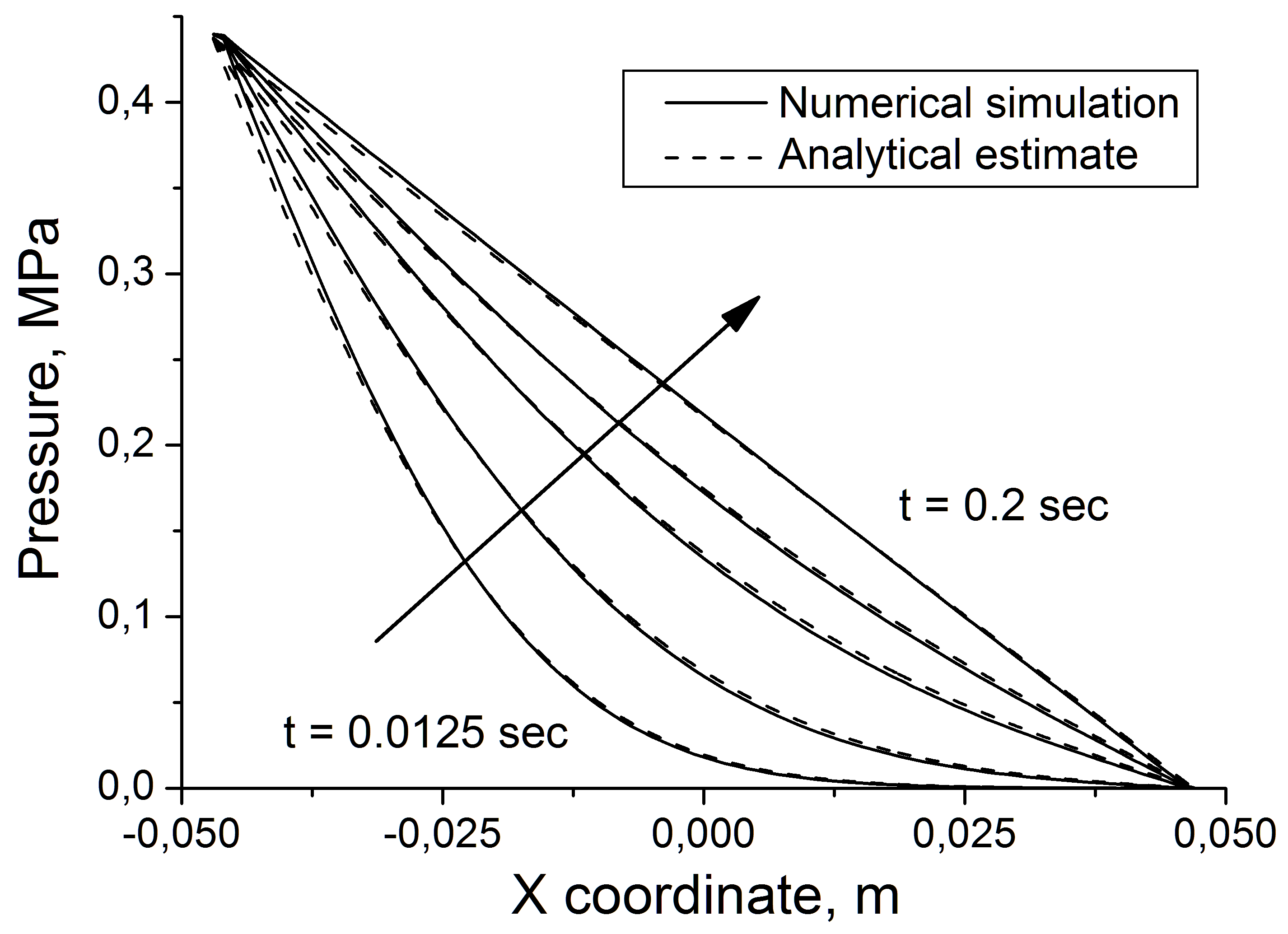 | 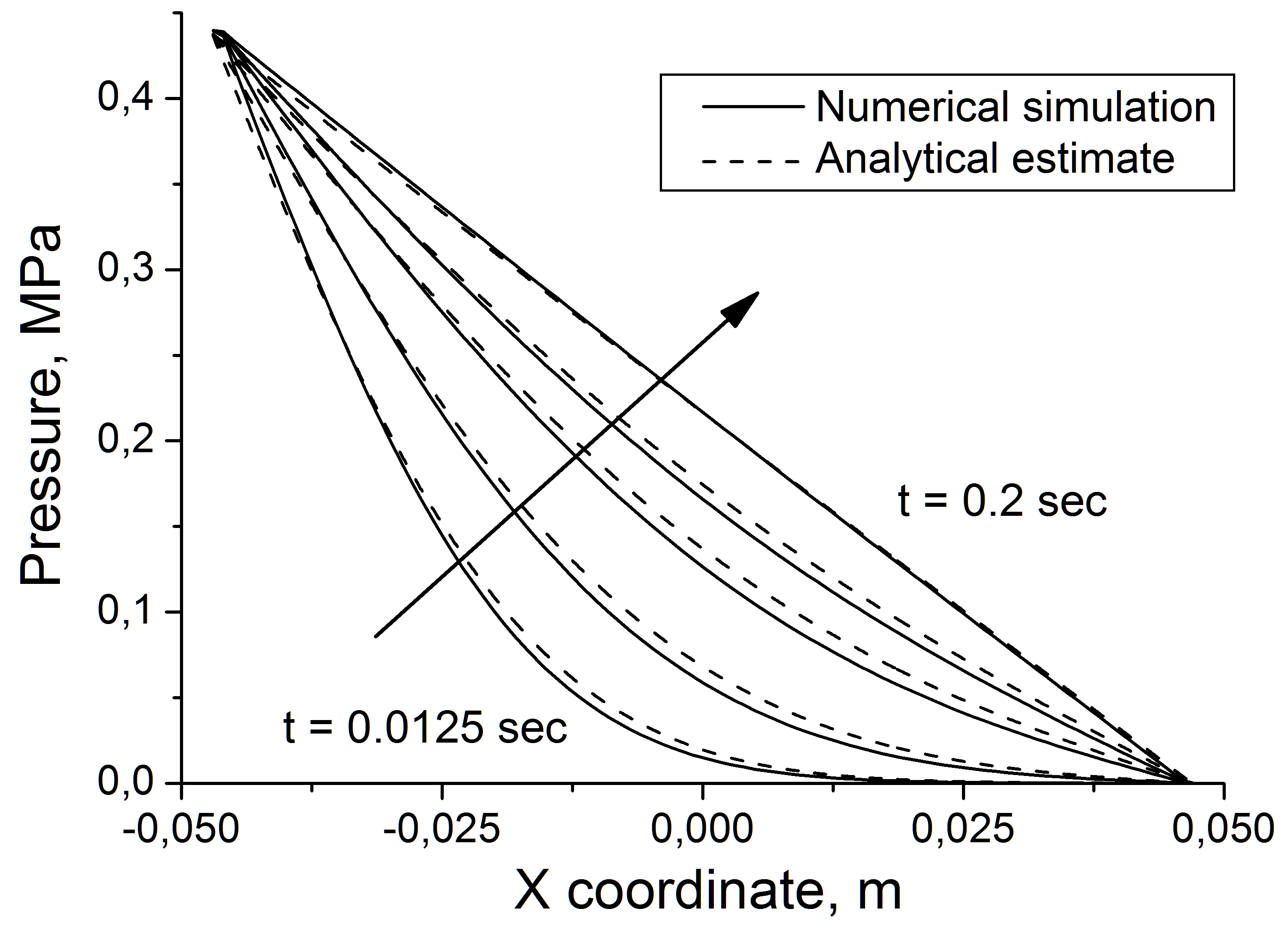 |
| --- | --- |
| a) | b) |
| **Figure S6**. Pore pressure distributions along the permeable sample at different points of time: a) constant porosity; b) pressure-dependent porosity. | |

The mechanical response of a fluid-saturated porous sample is governed by the following factors: 1) an elastic modulus of the solid skeleton; 2) presence of fluid in pores and its bulk modulus; and 3) fluid filtration from the solid skeleton to environment. The third factor provides a decrease in pore fluid pressure and, consequently, internal stress reduction in the solid skeleton. A result of this effect is a decrease in the reaction force of samples under compression.

The dynamics of stress-state variation in fluid-saturated samples during fluid discharge to environment was analyzed in the following numerical test. We modeled a rapid uniaxial deformation process of a permeable water-saturated elastic-brittle sample depicted in Supplementary Figure S5 () to an assigned strain with subsequent fixing of punches (the sample remained in the deformed state, see Supplementary Figure S7a). Rapid deformation here means sample loading to an assigned axial strain during the time interval which is much shorter than the characteristic time of fluid filtration through the sample cross section.

After sample deformation was finished and punches were fixed, reaction force on the punches gradually decreases (Supplementary Figure S7b) as a result of fluid outflow to the environment. Neglecting the dependence of porosity on pore pressure, it can be assumed that the decrease of sample pressure on punches occurs exponentially. In this case, the initial pressure corresponds to the pressure induced by the deformed water-saturated sample in the absence of filtration, and the final pressure corresponds to the pressure acting on punches from a “dry” sample.

| 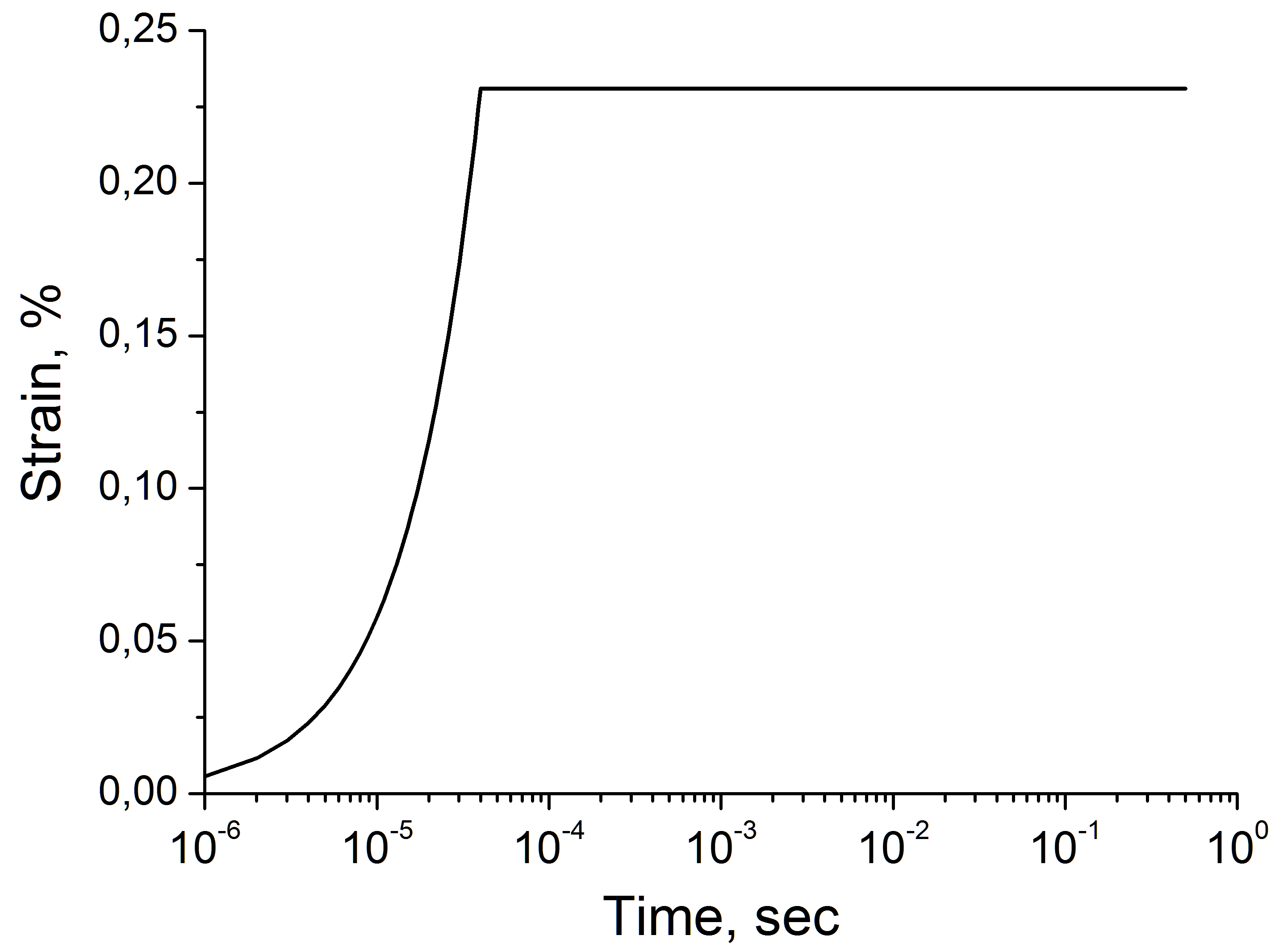 | 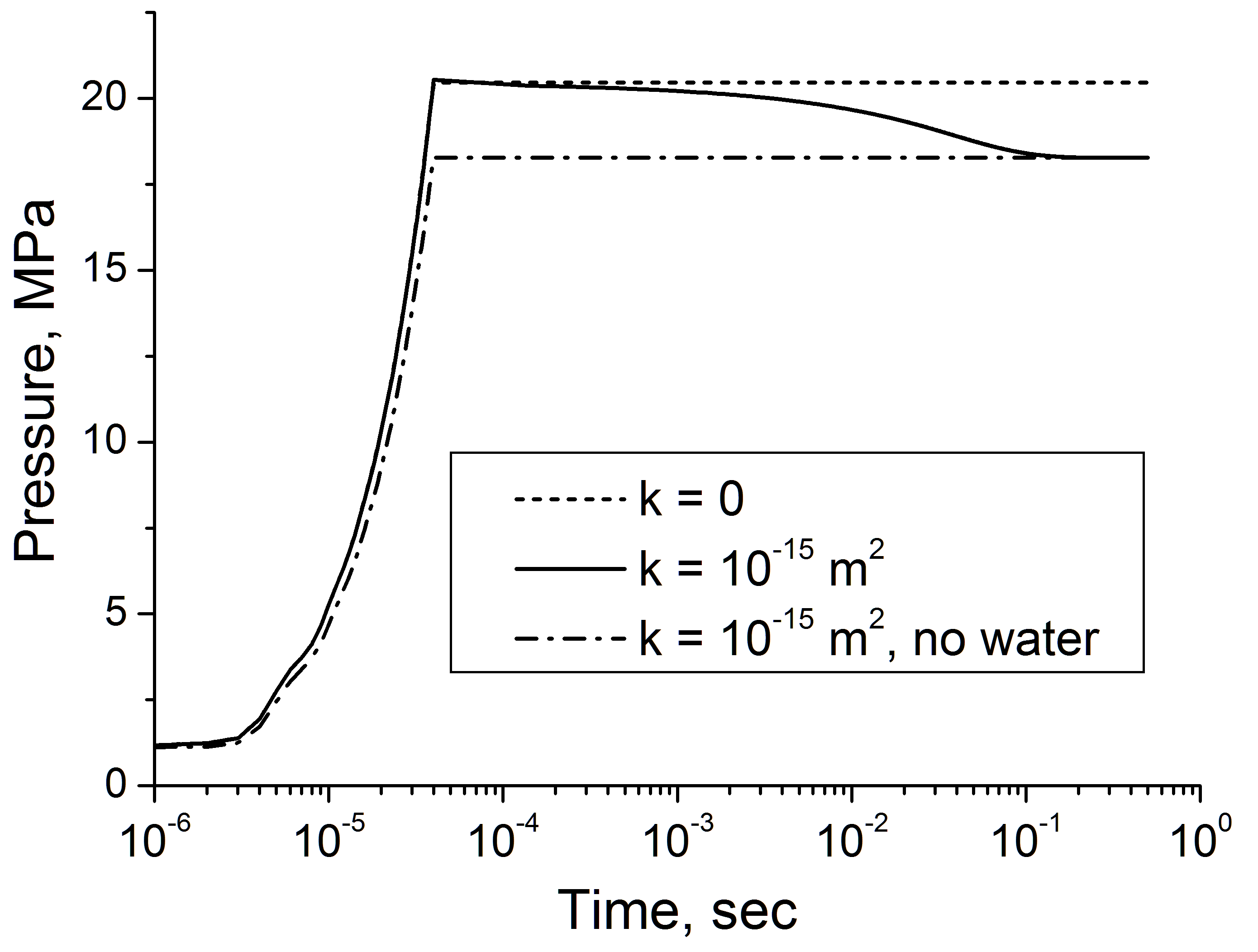 |
| --- | --- |
| a) | b) |
| **Figure S7**. The dependence of sample axial strain on time (a): the dependences of sample reaction force on time (b). Initial fluid pressure in pores of water-saturated samples was assumed to be zero. | |

The above assumptions were also used to model uniaxial compression of a water-saturated sample with zero permeability $k=0$ that leads to the absence of fluid transfer between micropores and its discharge to the environment, and a sample with no fluid in its pores. It is seen from Supplementary Figure S7b that the numerical simulation results fully correspond to the above-described form of the time dependence of the reaction force of the deformed sample.

Detournay et al. showed [5] that for a loaded sample of fluid-saturated poroelastic material the following condition holds true after the transient process governed by fluid discharge to the environment was terminated:

$\zeta=a\theta$, (S.5)

where $\zeta$ is the change of fluid concentration in a porous medium, $\theta$ is the bulk strain of a unit volume of the medium and $a=1-K/K_{s}$. In the above-considered case, the value of $\zeta$ is approximately defined as:

, (S.6)

where $m_{0}$ is the initial amount of fluid in the pore volume of the sample, and $m_{\infty}$ is the amount of fluid in the sample after fluid discharge to the environment was terminated. The residual volume of fluid in the sample was $\zeta\approx-4.1\cdot10^{-4}$. The bulk strain of the sample after the transient process termination was $\theta\approx-6.32\cdot10^{-4}$. The ratio $\zeta/\varepsilon=0.648$ corresponds with good accuracy to the assigned value of coefficient $a=1-K/K_{s}\approx0.649$.

The results of the described tests substantiate the adequacy of the developed model of fluid-saturated porous medium and its applicability to studying the mechanical response and strength properties of fluid-saturated brittle materials.

1. **The influence of discrete element size and packing type on the dependence of shear strength of the shear band on the parameter *A_xy_***

To study mesh dependence of the obtained numerical results (namely the profile and extremum points of the dependence shear strength of mechanically constrained shear band on the parameter *A_xy_*) we changed both discrete element size and packing type (Supplementary Figure S8). We modeled the sample by the ensemble of regularly packed equally sized discrete elements. We considered two types of packing: close packing and square packing. Simulation results have shown that the element size 5⋅10^-4^ m is small enough (further decrease in element size doesn’t lead to noticeable quantitative difference in strength value). Change in packing type leads to moderate quantitative change in shear strength value (within 15%) but not to change in the curve profile. Moreover, the positions of local minima of strength (*A_xy_*~1.5 GPa) are the same for both packing type, while the positions of local maxima of strength (*A_xy_*~0.5 GPa) differ less than 1.5 times.

| 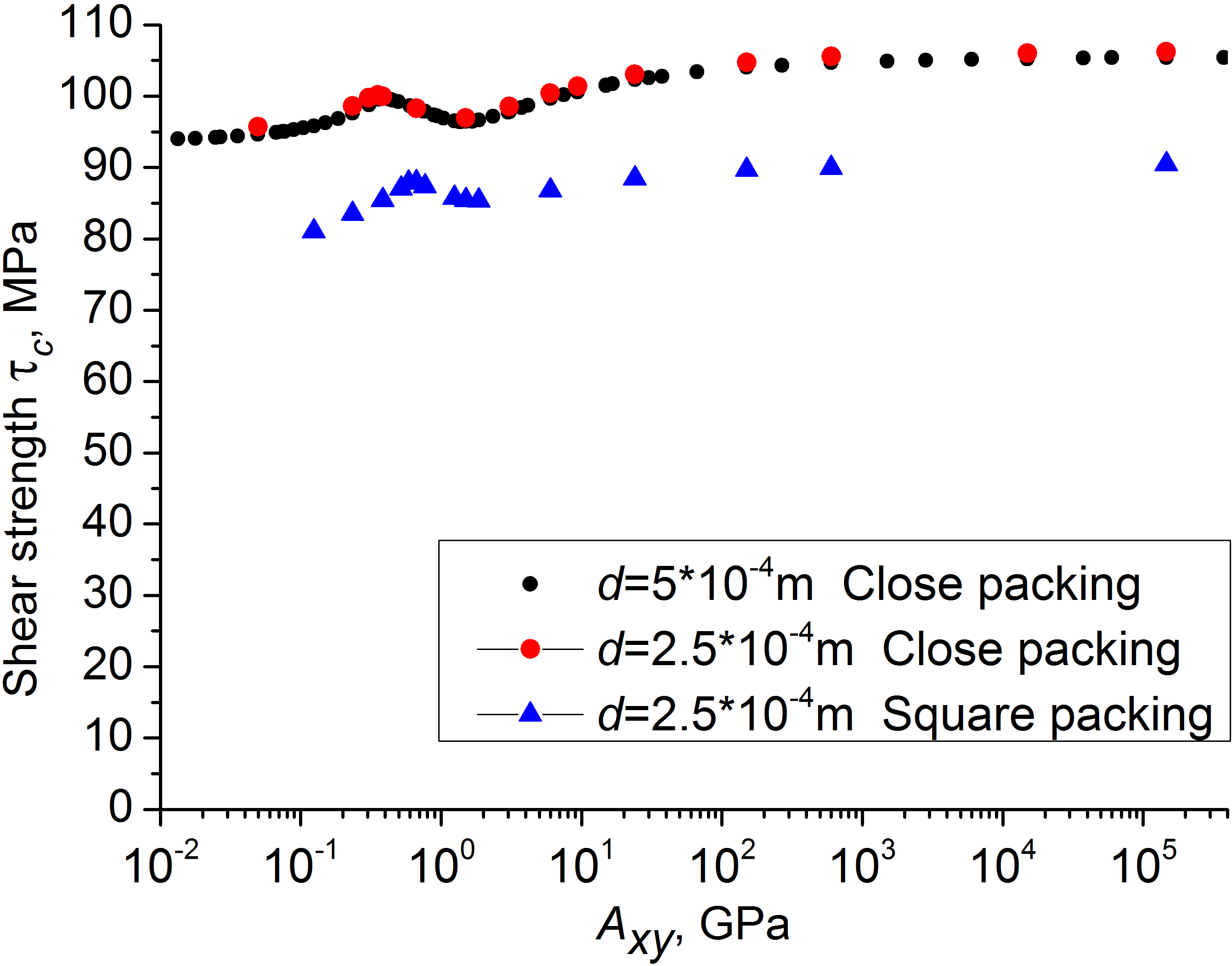 |
| --- |
| **Figure S8**. A typical numerically derived dependences of the shear strength τ*_c_* of the shear band on the parameter *A_xy_* for hydraulically isolated samples (*h*/*H* = 0.05, $P_{pore}^{0}=0.5\left\vert\sigma_{mean}^{0}\right\vert=$17.5 MPa). Three sets of points corresponds to different sizes of discrete elements and different types of packing of discrete elements. |

The demonstrated agreement between numerical results obtained using different types of packing of discrete elements ensures the correctness of the simulation method.

1. **Analytical estimation of the amplitude τ_3_ of the third contribution to shear strength of fluid saturated shear band**

Special numerical study at different values of initial pore pressure $P_{pore}^{0}$ and the same value of initial mean stress $\sigma_{mean}^{0}$ has shown that the amplitude τ_3_ of the third contribution to shear strength of fluid saturated shear band is proportional to the value of pore pressure $P_{pore}^{\max}$ in the “fully drained” state of the shear band (*A_xy_*→0) at the moment of failure: $\tau_{3}=A\cdot P_{pore}^{\max}$. The coefficient of proportionality *A* can be roughly estimated from the failure criterion (in our case from the Drucker–Prager criterion):

$1.5\left(\lambda-1\right)\left(\sigma_{mean}+P_{pore}\right)+0.5\left(\lambda+1\right)\sigma_{eq}=\sigma_{c}$,

where $\lambda=\sigma_{c}/\sigma_{t}$ is the strength ratio of “dry” material under uniaxial compression (σ*_c_*) and tension (σ*_t_*).

At *A_xy_*→0 pore pressure is nearly homogeneously distributed in the sample during the entire course of shear deformation (Figure 4d in the paper). Let us make the assumption that this is valid not only at *A_xy_*→0 but in the whole region III (Figure 2b in the paper). In this case we can roughly assume that the difference in strength between samples at “boundary points” *A_xy_*→0 and *A_crit_*_2_ is determined only by the difference in the “final” values of pore pressure $P_{pore}^{failure}$ (at the moment of failure criterion satisfying). We know that $P_{pore}^{failure}$=0 at *A_xy_*=*A_crit_*_2_ (where the third contribution to strength of the shear band approaches zero) and increases as *A_xy_* decreases. $P_{pore}^{failure}$ reaches maximum value $P_{pore}^{\max}$ at *A_xy_*→0 (where the third contribution to strength of the shear band approaches its maximum value τ_3_). Using the above mentioned assumption we can estimate the difference in strength between these two “boundary points” as determined only by difference in $P_{pore}^{failure}$:

$1.5\left(\lambda-1\right)\Delta P_{pore}^{failure}\approx1.5\left(\lambda-1\right)P_{pore}^{\max}\approx\Delta\sigma_{c}$, (S.7)

The threshold value σ*_c_* corresponds to the strength under uniaxial compression. Under uniaxial compression it is numerically equal to the limiting value of equivalent stress. At the same time it is known that under the condition of pure shear the equivalent stress is proportional to the shear stress with proportionality coefficient $\sqrt{3}$. So, we can conditionally associate compressive strength σ*_c_* with some shear strength τ*_c_*: $\sigma_{c}\to\sqrt{3}\tau_{c}$. This leads to the following approximate expression:

$1.5\left(\lambda-1\right)P_{pore}^{\max}\approx\sqrt{3}\Delta\tau_{c}$, (S.8)

The value of this “equivalent” τ*_c_* approaches maximum value at ” *A_xy_*→0 and zero value at *A_xy_*→*A_crit_*_2_. It is logical to associate it with the third contribution to shear strength of the shear band:

$\Delta\tau_{c}\to\tau_{3}\approx0.5\sqrt{3}\left(\lambda-1\right)P_{pore}^{\max}$, (S.8)

Figure 6a in the paper shows three sets of numerically obtained points approximated by the expression (5) in the paper. These sets of points correspond to hydraulically isolated samples (impermeable external boundaries) at two different values of initial pore pressure and the samples with perfectly permeable external boundaries. Amplitudes τ_3_ of the third contribution in Eq. (5) for all three cases presented in Figure 6a were calculated using (S.8) and the values of $P_{pore}^{failure}$ at *A_xy_*=10^-2^ GPa (we suppose that it is quite close to $P_{pore}^{\max}$):

1) hydraulically isolated samples at $P_{pore}^{0}=0.5\left|\sigma_{mean}^{0}\right|=$17.5 MPa: $P_{pore}^{failure}$≈ 4.9 MPa, τ_3_≈8.5 MPa.

2) hydraulically isolated samples at $P_{pore}^{0}=0.2\left|\sigma_{mean}^{0}\right|=$7 MPa: $P_{pore}^{failure}$≈ 0.09 MPa, τ_3_≈0.16 MPa.

3) perfectly permeable boundaries at $P_{pore}^{0}=0.5\left|\sigma_{mean}^{0}\right|=$17.5 MPa: $P_{pore}^{failure}$≈ 17.5 MPa, τ_3_≈30.3 MPa.

These estimated values of τ_3_ are used in the approximating curves in Figure 6a in the paper.

**References**

1. Psakhie, S.G. *et al.* A mathematical model of particle–particle interaction for discrete element based modeling of deformation and fracture of heterogeneous elastic–plastic materials. *Eng. Fract. Mech.* **130**, 96-115 (2014).
2. Stefanov, Yu. P. Deformation localization and fracture in geomaterials. Numerical simulation. *Phys. Mesomechanics*. **5**(5-6), 67-77 (2002).
3. Psakhie, S.G., Dimaki, A.V., Shilko, E.V. & Astafurov, S.V. A coupled discrete element-finite difference approach for modeling mechanical response of fluid-saturated porous material. *Int. J. Numer. Meth. Engng.* **106**, 623-643 (2016).
4. Loytsyanskii, L.G. Mechanics of Liquids and Gases. Pergamon-Press, 1966.
5. Detournay, E., Cheng, A.H.D. “Fundamentals of poroelasticity”. Chapter 5 in Comprehensive Rock Engineering: Principles, Practice and Projects, Vol. II, Analysis and Design Method, ed. C. Fairhurst. Pergamon Press, 1993; 113-171.
